# Supplementary material for: Advancing aphasia psychological care in Ireland: A participatory study with people with aphasia and clinicians
Source: PLoS One. 2026 Apr 20;21(4):e0331427. doi: 10.1371/journal.pone.0331427 (PMC13095103; doi:10.1371/journal.pone.0331427)
Supplement: S2 File — (PDF) [file pone.0331427.s002.pdf]

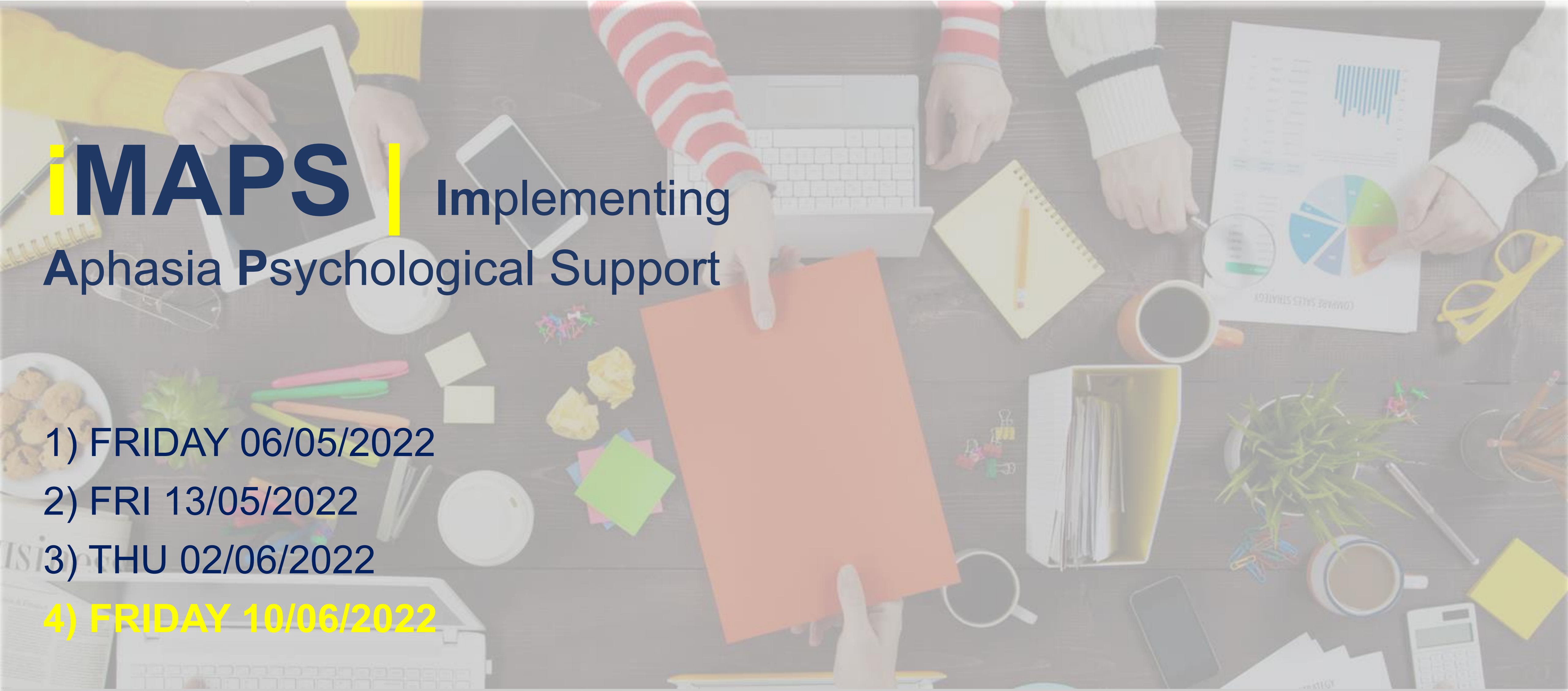

iMAPS | Implementing Aphasia Psychological Support

1) FRIDAY 06/05/2022
2) FRI 13/05/2022
3) THU 02/06/2022
4) **FRIDAY 10/06/2022**

"Live record" Co-design sessions

12 – 1.15 pm

Recap | sessions 1 – 4

- ✓ ISSUES + CONSIDERATIONS 6.5.22
- ✓ SCOPE 13.5.22
- ✓ “IDEAL” PRINCIPLES 13.5.22
- ✓ PPI feedback 30.5.22
- ✓ Aphasia spc considerations in Irish context 2.6.22 and 10.6.22
- ✓ What are priorities / where to start? 10.06.2022
- ✓ Stakeholders & dissemination 10.06.2022
- ✓ Close

Recap | sessions 1 – 4

✓ ISSUES + CONSIDERATIONS 6.5.22

- ↓ Access + variation
- ↓ HCP understanding of support needs
- ↓ Structural support for HCP
- HCP training
- Different needs @ different stages
- Signposting ≠ the destination

✓ SCOPE 13.5.22

✓ "IDEAL" PRINCIPLES 13.5.22

1) A better pathway

2) Team structures, resourcing

3) Training + support for clinicians

4) Focus of care

✓ PPI feedback 30.5.22

✓ Aphasia spc considerations in Irish context 2.6.22 and 10.6.22

1. Implementation levers + barriers
2. Person-centred + integrated care
3. Families
4. Who do we need?
5. Involving clinicians + PLA
6. Specific interventions (L1-4)
7. Support & training for clinicians
8. Transdisciplinary working

Recap | sessions 1 – 4

✓ **What are priorities / where to start? 10.06.2022**

- Adapting health communication environment
- Service design
- Dissemination & impacting policy
- Family support
- Self-management & Peer support
- "Alternative modalities"

Clinician knowledge & support

- Aphasia awareness across continuum of care
- Clinician support & self-care
- Self-directed / online
- Pre-qualification training
- Interdisciplinary training / co-learning

✓ **Stakeholders & Dissemination 10.06.2022**

✓ **Close**

SESSION 1
FRI 6 MAY 22

Today

1. Meet each other
2. About this study + co-design sessions
3. Background context + information
4. Discussion

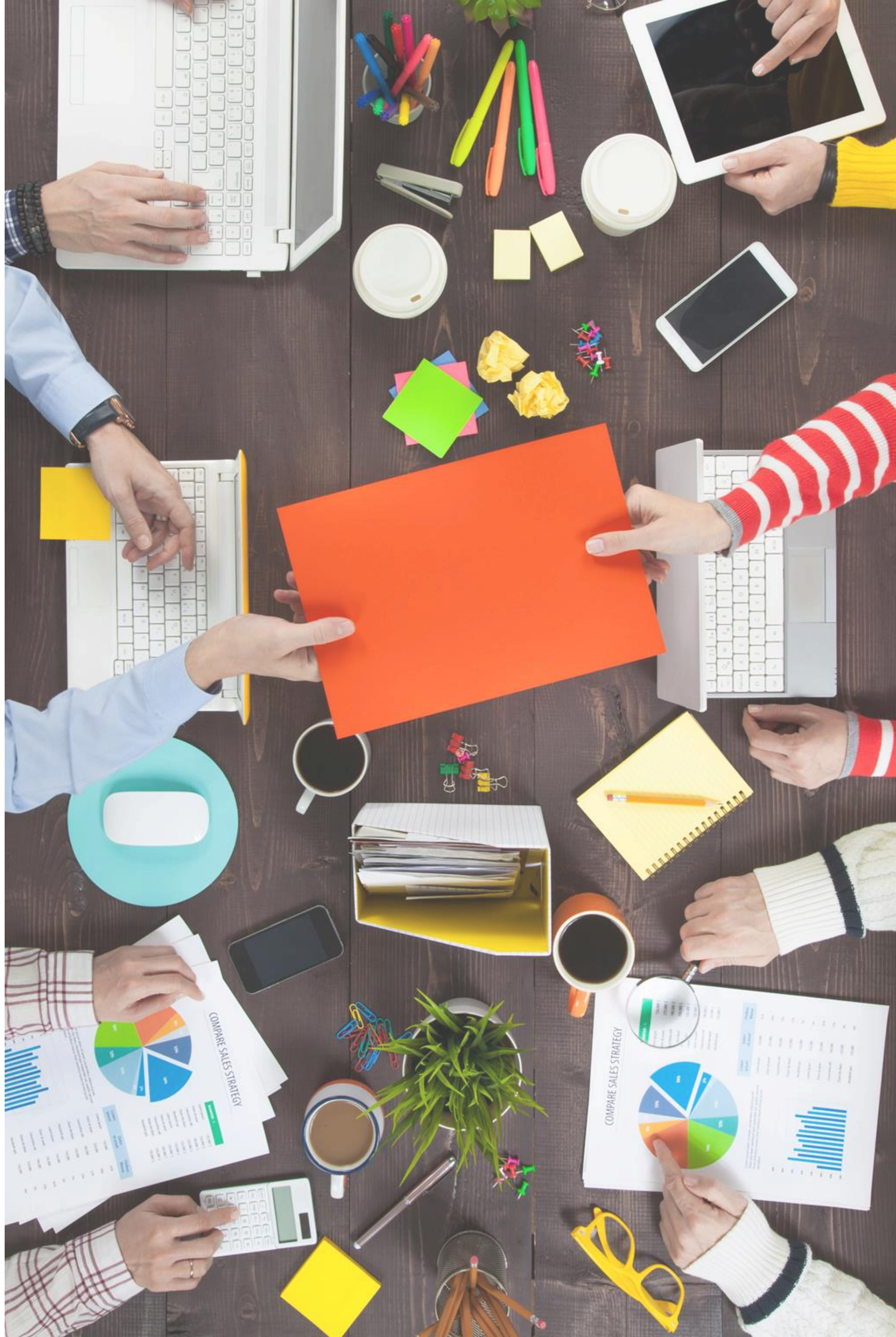

Today

1. **Meet each other**
2. About this study + co-design sessions
3. Background context + information
4. Discussion

- ✓ Role + work setting
- ✓ APS experience / interests
- ✓ Why you are taking part in co-design sessions

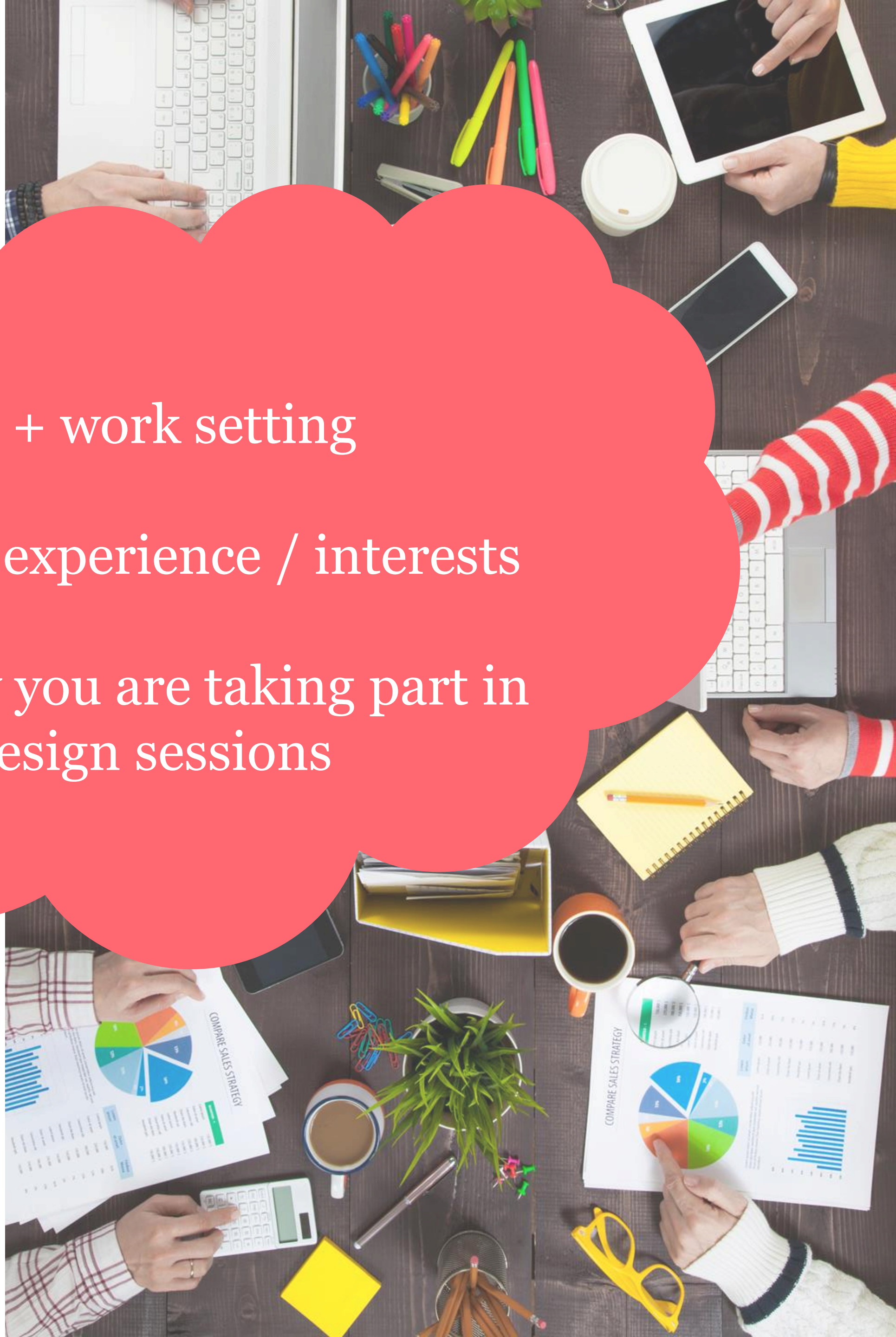

Today

1. Meet each other

2. **About this study + co-design sessions**

3. Background context + information

4. Discussion

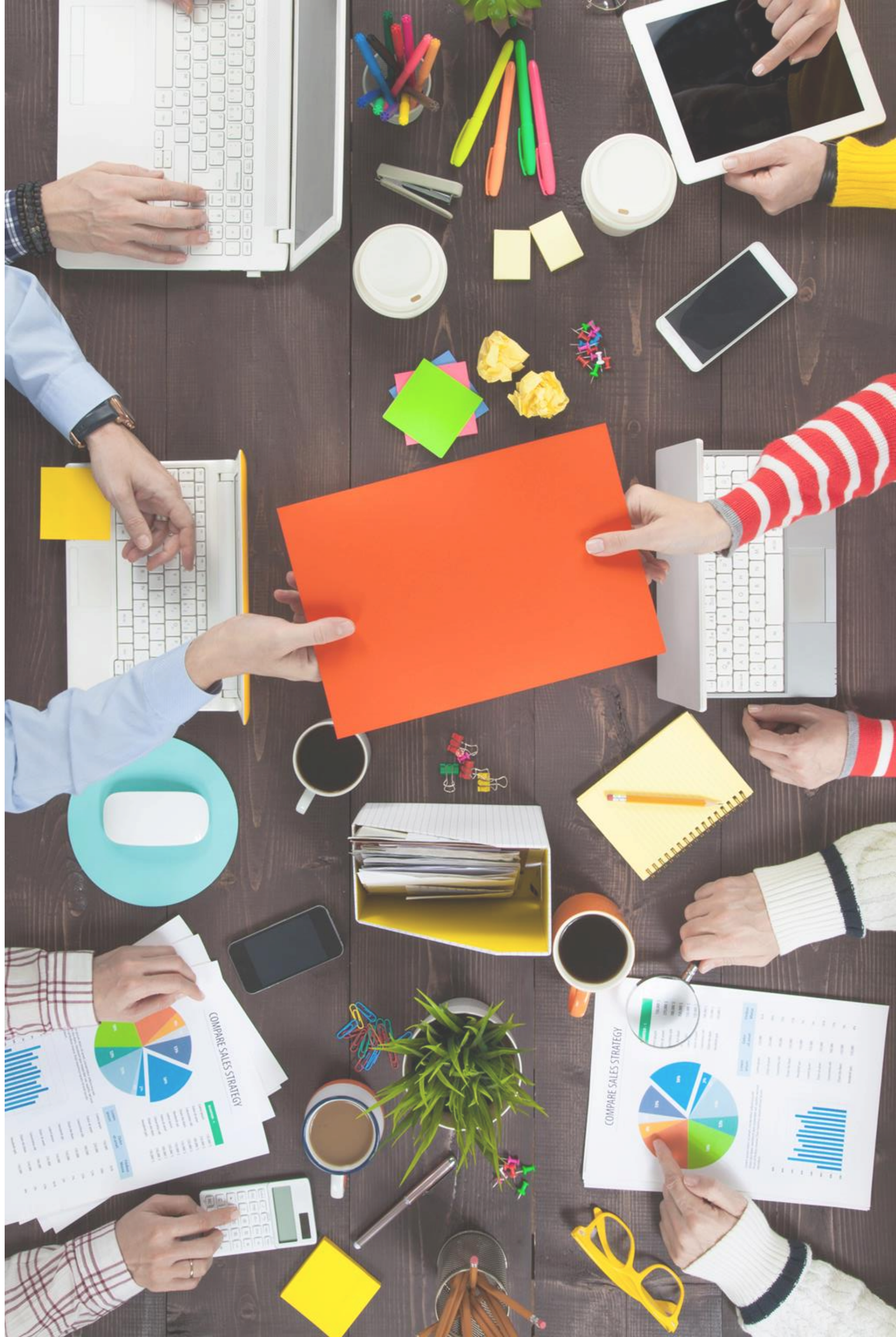

About this study + co-design sessions

- iMAPS funded by UL Faculty of Education & Health Sciences.
- Will generate evidence + ideas on enhancing aphasia support + will inform future funding applications.
- Participatory Health Research + Implementation Science.
- All affected by the change should be involved in its **local adaptation, implementation and ownership**.

- Developing evidence pragmatically – taking evidence + studying how it can be adapted + embedded effectively in local contexts *(e.g., changing processes, ways of working, structural support, policies, funding, outcomes, acceptability etc.)*

About this study + co-design sessions

⇒ **PPI and Clinician Co-design sessions**

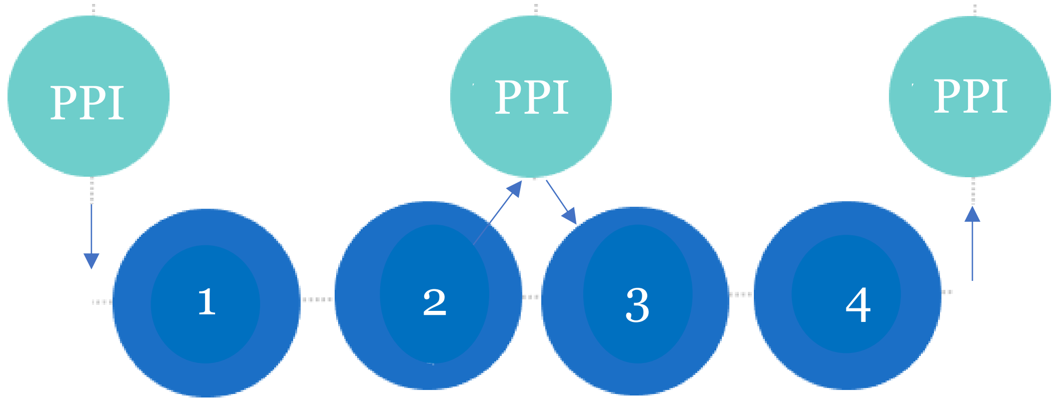

Clinician Co-design sessions

Initial discussions around

adapting + implementing

a stepped / integrated model

of aphasia care in Ireland.

Co-design purpose + format

Consent and ethical issues

- Research Ethics approval.
- We will treat everyone's contributions respectfully and confidentially.
- Sessions will be recorded to ensure we haven't 'missed' anything.
- Recordings will be deleted soon after meetings.

- If you cannot continue you can do so at any time, and you do not need to give a reason.
- Do not feel obliged to answer any questions that you are uncomfortable with.
- You can contact Molly or a 3rd party (see ethical consent form for details) if you have any concerns.

(brief) background context + information

- **Psychological problems are common.**
- Evidence that **treatment is effective.**
- **'Living well' with aphasia is promoted via:**
  - Responsive access to **SLT, informational, emotional, liaison and family support** at all stages.
  - Opportunities for **meaningful participation**.
- **Under-included in stroke trials – care inequities.**

- **Shortcomings in aphasia care.** Multi-level reasons.
- **Fragmentation** in the way services are set up; **no integrated care pathway.**
- Lack of consistent **access + availability**.
- **Lack of** confidence, training, interprofessional working + top-down **support for clinicians.**

MOMENTUM / APPETITE FOR CHANGE?

- Health policy imperatives -
  - Person-centred, integrated care.
  - Personally-meaningful health outcomes.
  - Perspectives, inclusion, PPI.

- Interdisciplinary SEA Community of Practice.
- National Stroke Strategy; IHF Council on Stroke.

- Additional momentum in aphasiology in **adapting the interdisciplinary stepped psychological model (stroke)** to make it relevant and evidence-based for aphasia.

- **The adapted model** specifies aphasia-specific therapeutic interventions, the role of SLT, and much potential for reciprocal interdisciplinary support, mentorship & collaboration.

- We will talk in more detail about this model at session #2.

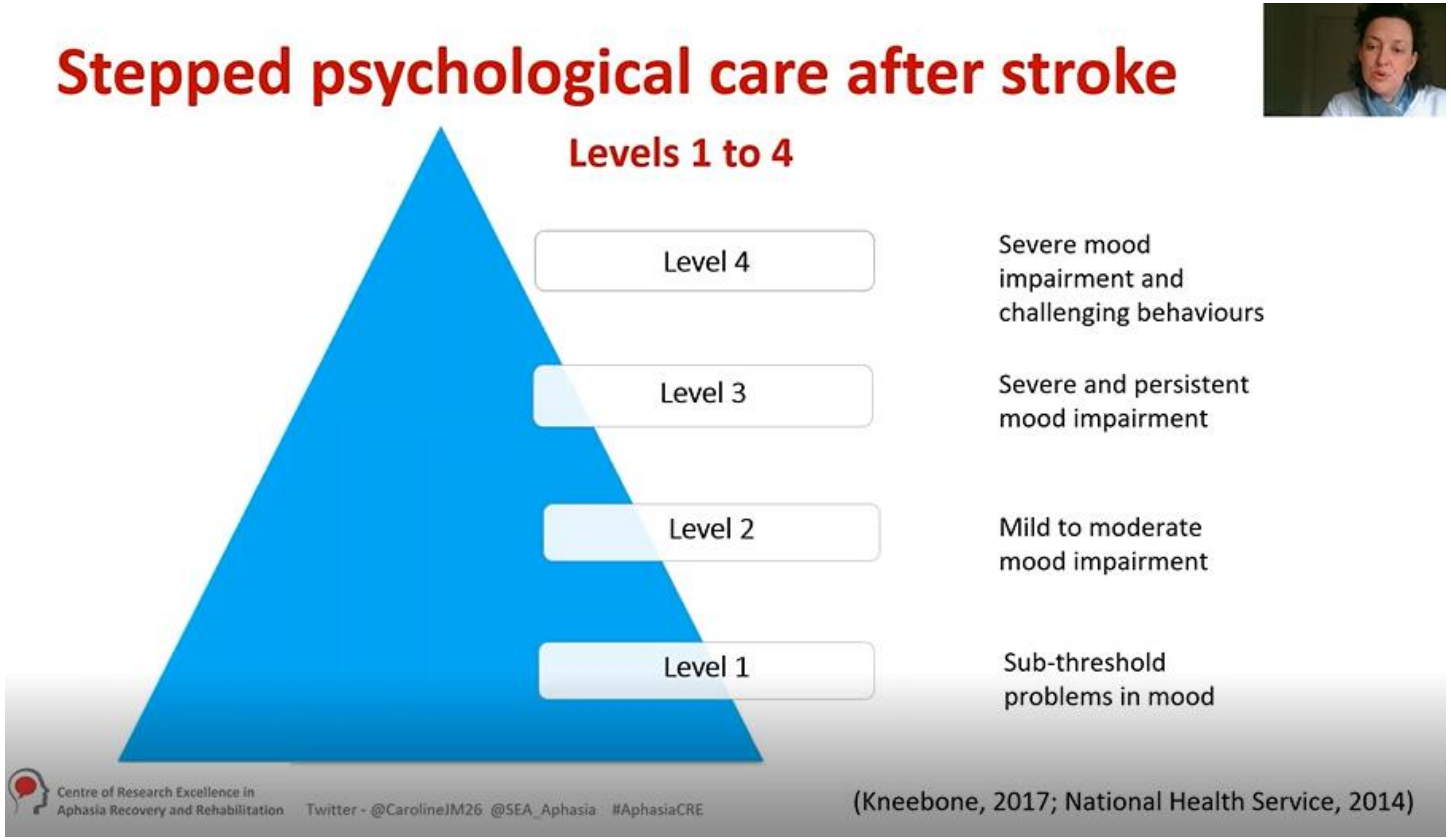

This slide includes a still from Dr Caroline Baker's excellent SEA 2 presentation.

I will circulate a link.

Today

1. Meet each other

2. About this study + co-design sessions

3. Background context + information

4. Discussion

Consensus that background information 'rings true'.

Various issues identified – these are presented thematically.

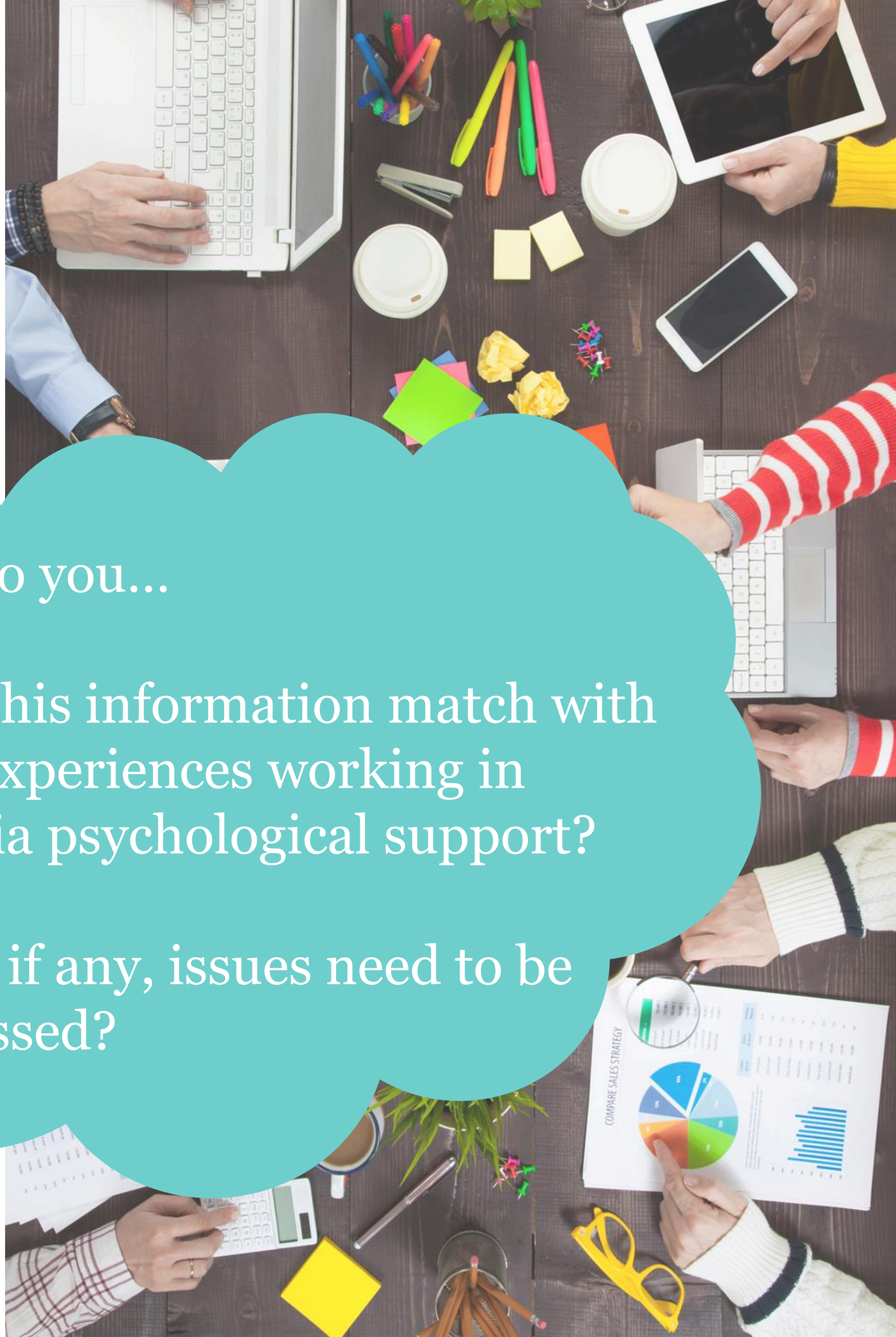

Over to you...

Does this information match with your experiences working in aphasia psychological support?

What, if any, issues need to be addressed?

Recap

Session 1: 06 May 2022

Contributions in response to the background information, project purpose + evidence.

Consensus + comments themed.

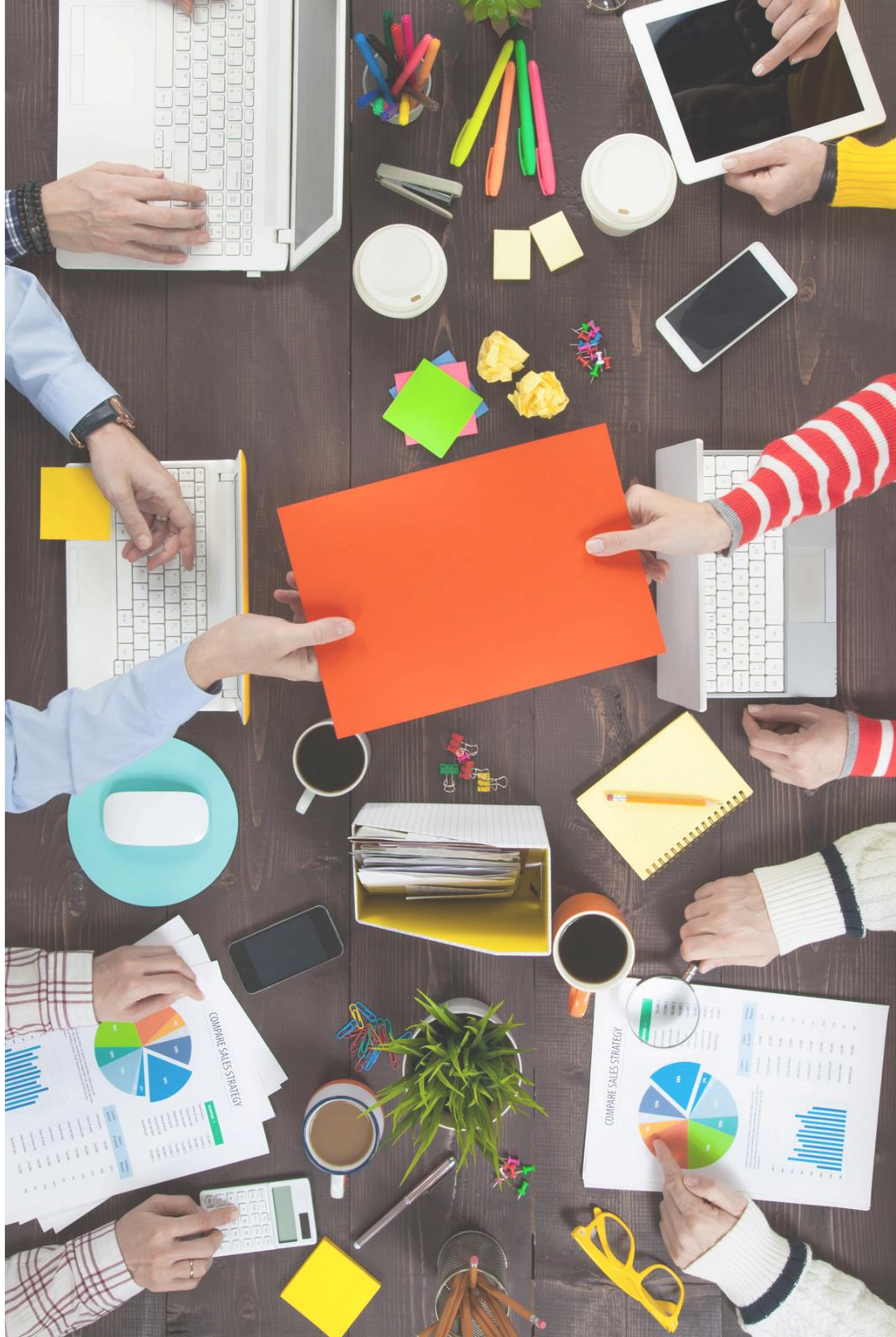

Issues + considerations

(for record only – skip through for summary themes)

Not sure NSS changes will translate to community.

No specialist pathway of care – fall under general ‘umbrella’.

Adult MH care configured differently. MH secondary to ABI very different. Often not discerned, even among PSY.

PLL in acute setting (not Psych)

talk therapy enough - ignorance re ideal care (counselling enough)

Rehab (only last few months access to psy - has bn incredible)

Also ? aphasia as barrier to accessing services (can't speak, not suitable)

SLT advocating on their behalf.

SLT - conversations emotional. ++learning letting client be. Not trying to do / fix. Permission to let them be. through training. Needed it.

distressing to speak with someone distressed etc. human instinct to avoid.

F/back from acute pts - suddenly wake up (in nappies); feels foreign to them to be crying all the time & to require psych input suddenly.

Issues + considerations

(for record only – skip through for summary themes)

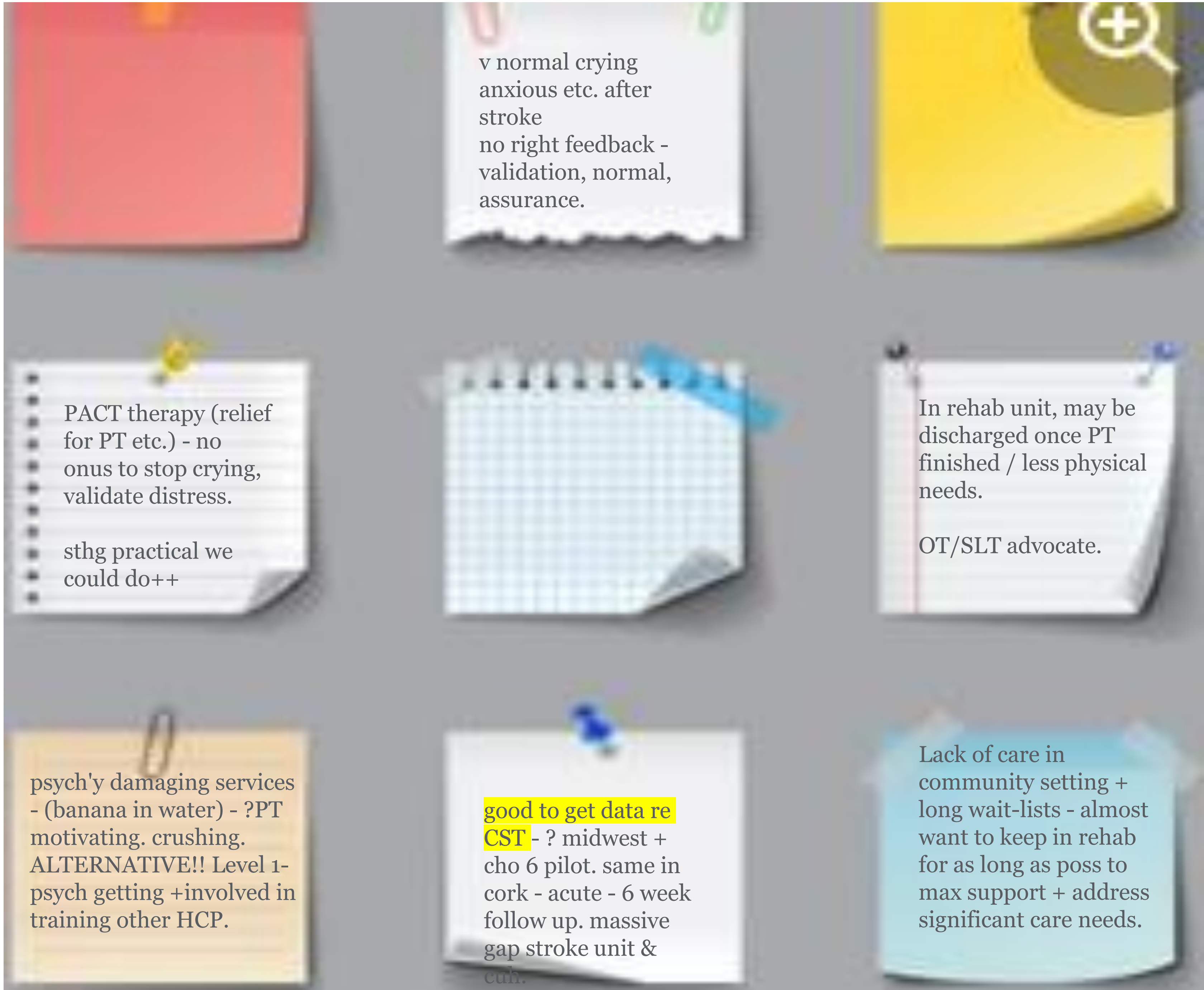

v normal crying anxious etc. after stroke
no right feedback - validation, normal, assurance.

PACT therapy (relief for PT etc.) - no onus to stop crying, validate distress.

sthg practical we could do++

In rehab unit, may be discharged once PT finished / less physical needs.

OT/SLT advocate.

psych'y damaging services - (banana in water) - ?PT motivating. crushing. ALTERNATIVE!! Level 1- psych getting +involved in training other HCP.

good to get data re CST - ? midwest + cho 6 pilot. same in cork - acute - 6 week follow up. massive gap stroke unit & cuh.

Lack of care in community setting + long wait-lists - almost want to keep in rehab for as long as poss to max support + address significant care needs.

Issues + considerations

(for record only – skip through for summary themes)

Nervous re reliance on professionals as solution. never enough professionals - in any sector. placing hope in potential access NOT the way forward.

It's a Level 4 understanding of disability (relying on professional input for living lives).

More profs not the answer - true role not to sit in clinical room with client and "dispense the magic" = cul de sac - disseminate WHAT WORKS.

The support networks (family, friends) need the "magic" - we are sign-=posters not the destination.

People want to 'get back' to how they were before in rehabilitation. But working on (medical) recovery may rob potential from idea of acceptance.

Constant pull of clinicians. – encouraging to "work harder" for improvement, but this is not the journey / experience / attainment for everyone.

Issues + considerations

(for record only – skip through for summary themes)

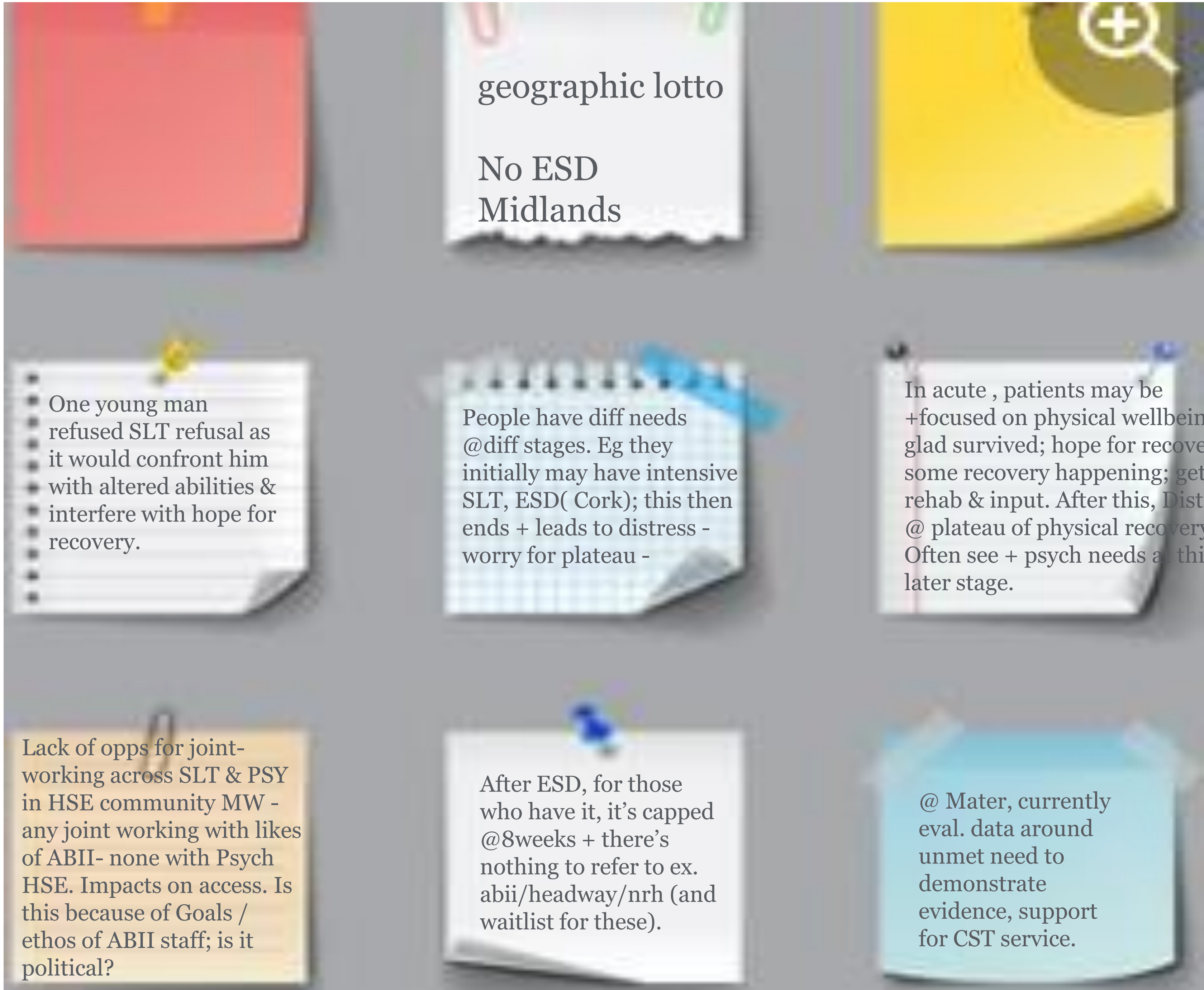

Acute care | ESD | I/P Rehab | CRT | HSE community | 3rd sector

Issues + considerations

↓ Access + variation

↓ HCP understanding of support needs

↓ Structural support for HCP

HCP training

Different needs @ different stages

Sign-posting ≠ the destination

Acute care | ESD | I/P Rehab | CRT | HSE community | 3rd sector

↓ Access + variation

- Inconsistent access, availability, configuration of health, psych support.
- No specialist pathway of care (fall under general 'umbrella').
- Geographical variation. (e.g., ESD midlands, CST only CHO3/6 - need data).
- Lack of onward CST / community support @ Mater (only option is to refer to 3rd sector / NRH but ++ waiting).

↓ HCP understanding of support needs

- Support for MH issues with ABI is very different from general MH care. Often not discerned, even by PSY.
- Perception that “talk therapy” is sufficient without understanding role of PSY.
- Improved physical ability and discharge from PT may mean rehab discharge.
- Psychologists not seeing people with aphasia (‘can’t talk).
- Damaging comments e.g. from PT (in the belief that they are motivating).

↓ Structural support for HCP

- Can be isolated in community even with SLT team.
- Lack of joint-working across SLT & HSE PSY. This impacts on access. Only possible with ABII etc.
- Is this due to goals/ethos of ABII +/- political issues?
- Lack of confidence NSS impact will translate to community.

Acute care | ESD | I/P Rehab | CRT | HSE community | 3rd sector

HCP training

- PACT as potential L1 intervention delivered by PSY to HCP incl. PT.
- SLT counselling training enables holding space, not needing to do / fix.

Different needs @ different stages

- Early in care patient may be more focused on physical improvement & on ongoing progress.
- May have sense of gratitude for surviving.
- Afterwards, may be distressing to discern plateau. Psych needs may be greater at this stage.
- E.g. of avoiding SLT as it interferes with hope.

Sign-posting ≠ the destination

- Under-staffing is a constant. There'll never be enough.
- We must reframe our emphasis on the importance of 'services' in supporting living well.
- There's a more fundamental question around how we view disability and how this feeds into services.
- Dominant focus: "level 4" understanding of disability which emphasis professional input for living lives.
- Focusing on medical recovery ('working harder' in rehabilitation) may impede personal recovery and re-engaging with life (as opposed to chasing a complete return to normal).
- We need to:
  - Disseminate 'what works'
  - Support the supporters (e.g., family, friends)
  - Emphasise personally defined recovery (within / without the context of services).

SESSION 2
FRI 13 MAY 22

Today

1. Agree scope

2. Brainstorm: Ideal service model

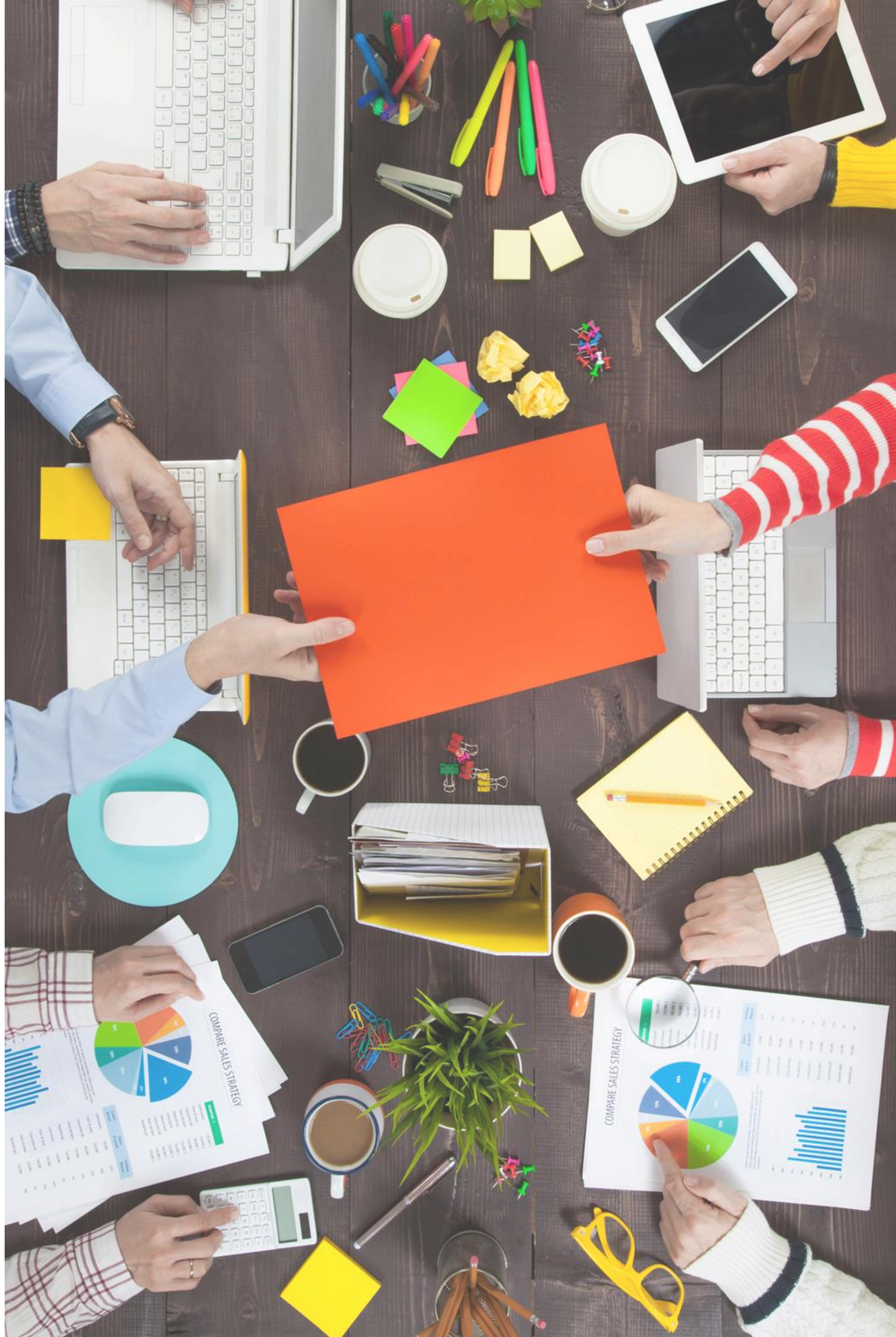

Discussion re 'psychological support' scope

"emotional support" so not seen as preserve of PSY. Broaden # people that can get involved.

Cognitive intervention should be in scope. Including general cognitive intervention (awareness, insight).

CBT - "core" intervention in context of emotional problems. Behavioural Activation is often starting point for depression w aphasia. In fact, BA is effective as cognitive element.

Including intervention for people with psychological distress, pre-existing mental health conditions

Therapeutic input (direct / indirect)

targeting improved emotional wellbeing and mood

CBT - targeting cognition, thoughts, behaviours, activities - all impact on wellbeing, feelings, being able to identify feelings, and whole-body work.

Behavioural activation interventions are applicable, accessible.

Again reference to the new Aphasia book which has lovely ideas re emotion, body work.

optimising wellbeing / social participation – not necessarily separate targets

potential for social prescription

social participation - Level 1 intervention?

Implementing aphasia emotional support | SCOPE agreed 13/05/2022

	In Scope	Out of Scope
**Patient Focus**	All individuals with aphasia secondary to ABI.<br><br>Includes pre-existing emotional / mood / psychiatric conditions.	• Progressive aetiologies including PPA.<br>• Acquired communication impairment with no aphasia.<br>• Family members as direct recipients of support.
**Pathway start / finish**	From event onset + following patient pathway throughout care continuum.	Excluding support prior to hospital admission & diagnosis.
**Emotional support**	• Therapeutic input (direct / indirect) targeting improved psychological wellbeing, mood and cognition.<br>• Social participation (eg., at Level 1)	
**Care settings & services**	Acute -> long-term support in the community. HSE & 3rd sector support organisations.<br><br>Acute care · ESD · I/P Rehab · CRT · HSE community · 3rd sector	

Implementing post-stroke aphasia psychological care | SCOPE agreed 13/05/2022

	In Scope	Out of Scope
**Patient Focus**	All individuals with aphasia secondary to ABI.<br><br>Includes pre-existing emotional / mood / psychiatric conditions.	• Progressive aetiologies including PPA.<br>• Acquired communication impairment with no aphasia.<br>• Family members as direct recipients of support.
**Pathway start / finish**	From event onset + following patient pathway throughout care continuum.	Excluding support prior to hospital admission & diagnosis.
**Emotional support**	• Therapeutic input (direct / indirect) targeting improved psychological wellbeing, mood and cognition.<br>• Social participation (eg., at Level 1)	
**Care settings & services**	Acute -> long-term support in the community. HSE & 3rd sector support organisations.<br><br>Acute care · ESD · I/P Rehab · CRT · HSE community · 3rd sector	

Today

1. Agree scope
2. Brainstorm: Ideal service model

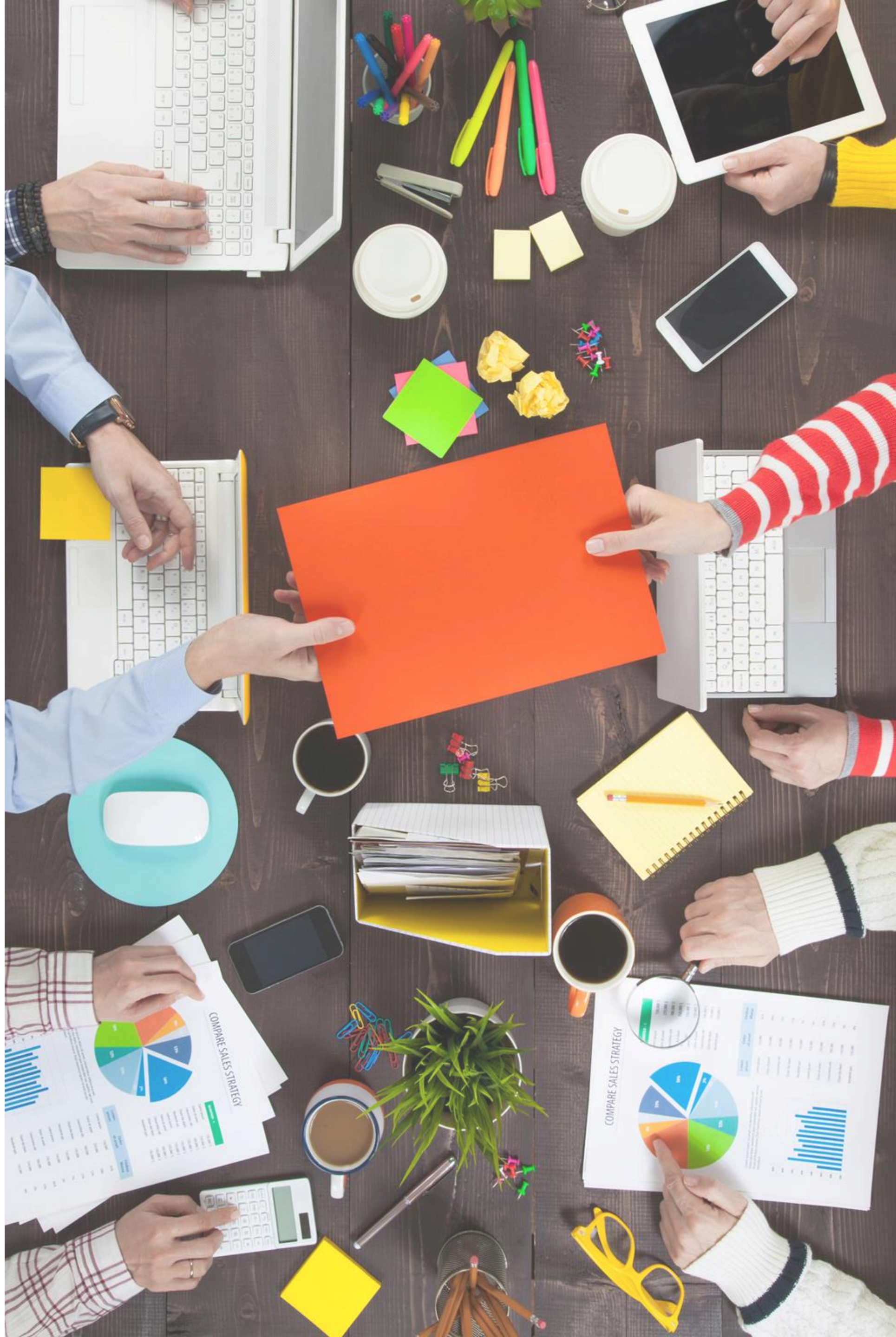

Question 1

What would an ideal model of emotional / psychological aphasia support look like?

Key principles

Training

Resourcing, Financing

Management and policy support

Team structures

Mentorship & Support

The type of care that is 'unlocked'

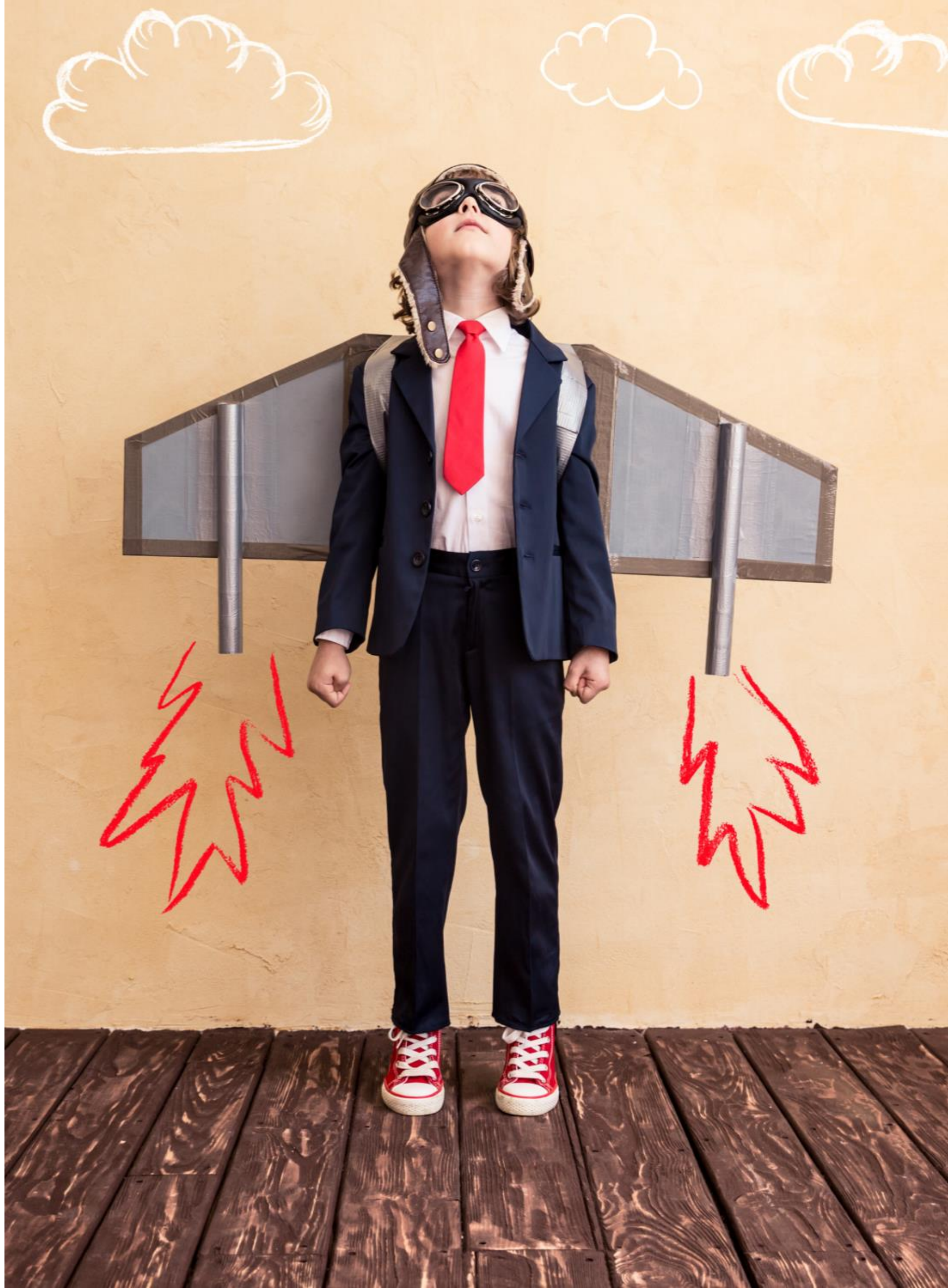

Key Principles

Psychological wellbeing after ABI is enhanced with "the basics" first principle interventions such as fatigue management, activity scheduling, physical exercise, mindfulness and participation in activities of daily living

Behavioral Activation is a key component of CBT, accessible to people with aphasia, and effective as a standalone therapy

Couples/ family/ systems work if needed

Working with the family as a unit is more effective than working with the individual alone

Families need support when someone has aphasia

Person and family centred care is crucial

Client centred care

Client Centred Care - What they need when they need it - not driven by our "agenda"

No matter where you live in Ireland, you are entitled to equality of access to effective therapy

Self referral capacity within the service

Service needs to be structured to flexible enough for service users to use service across stroke care continuum

Slaintecare principles more care at home or closer to home in communiities, also when needed and where wanted

Self-compassion and acceptance are as important as "recovery"

Psychological wellbeing comes from living the life of my choosing... the key principles are participation, relationships, competence (opp 4 activities in which competent - focus on fixing the things less competent) autonomy and dignity & respect

All rehabilitation should begin with the end in mind, which is defined by what is important to the person, not what is important to the clinician

Living well with aphasia

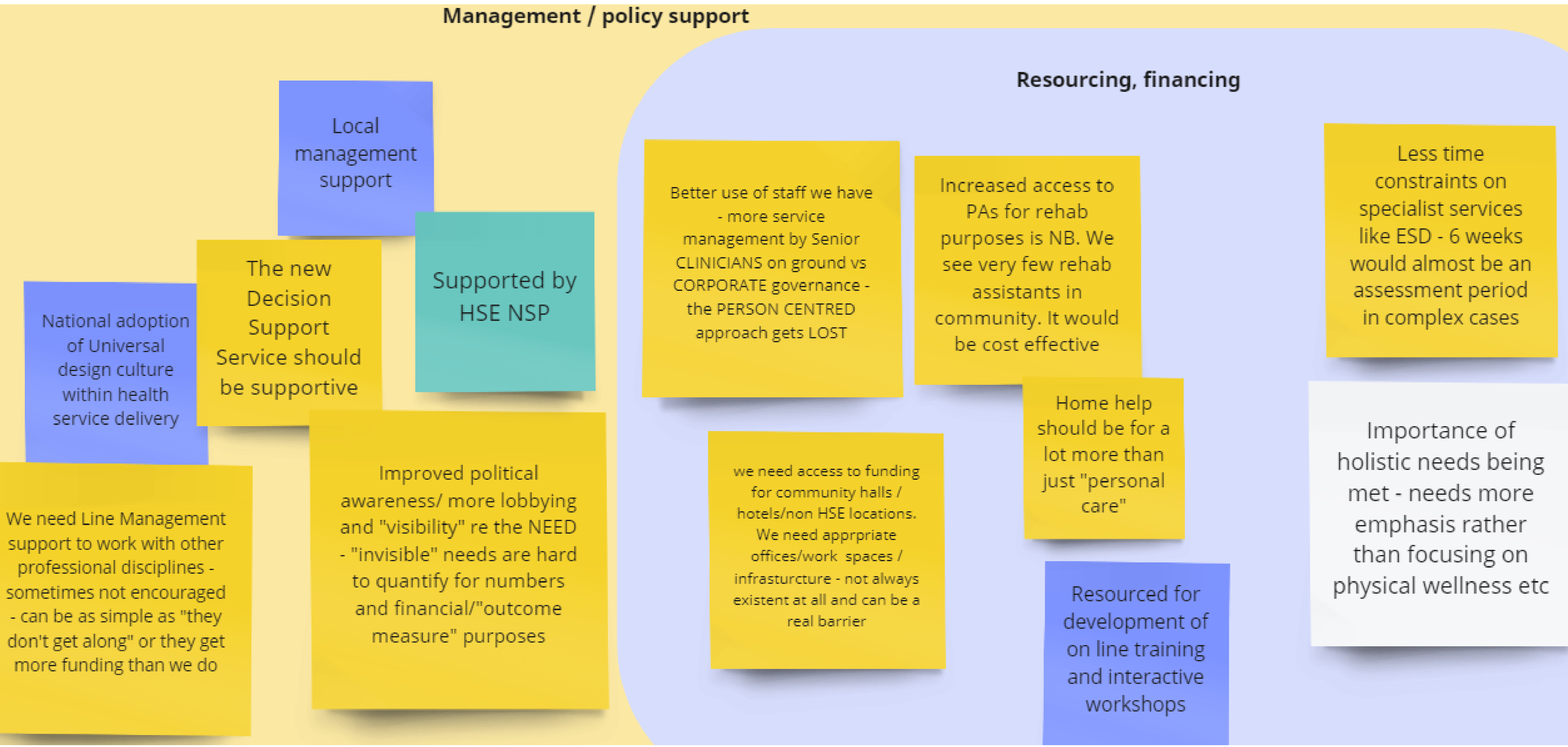
Management / policy support
Local management support
National adoption of Universal design culture within health service delivery
The new Decision Support Service should be supportive
Supported by HSE NSP
We need Line Management support to work with other professional disciplines - sometimes not encouraged - can be as simple as "they don't get along" or they get more funding than we do
Improved political awareness/ more lobbying and "visibility" re the NEED - "invisible" needs are hard to quantify for numbers and financial/"outcome measure" purposes
Resourcing, financing
Better use of staff we have - more service management by Senior CLINICIANS on ground vs CORPORATE governance - the PERSON CENTRED approach gets LOST
Increased access to PAs for rehab purposes is NB. We see very few rehab assistants in community. It would be cost effective
Less time constraints on specialist services like ESD - 6 weeks would almost be an assessment period in complex cases
we need access to funding for community halls / hotels/non HSE locations. We need apprpriate offices/work spaces / infrasturcture - not always existent at all and can be a real barrier
Home help should be for a lot more than just "personal care"
Importance of holistic needs being met - needs more emphasis rather than focusing on physical wellness etc
Resourced for development of on line training and interactive workshops

Training opportunities / knowledge needs

- Education for policy developers
- Workshops on Motivational Interviewing, supported communication, enabling healthy adjustment post ABI
- Community groups/day services for older adults
- more interdisciplinary training - typically a lot of our training is unidisciplinary BUT this is improving
- Client and their family/support network support & education
- Supported conversation is an important training framework
- Families need more support and training
- MDT Team ongoing CPD, support, transdisciplinary work
- HSE support with on line training similar to STARS training that would be mandatory for stroke HCPs
- GP Trainees
- Welfare officers
- Medics really need communication skills training full stop. For people with aphasia this need is even greater.
- Improved Consultant Physician and GP insight into psychosocial and invisible non overt life impacts of Stroke/ABI
- ?background, experience / suitability for roles, training

Team structures

Transdisciplinary working (80% of what we offer as professionals could be offered by any other professional on the team) rather than multi-disciplinary (each professional paddling their own canoe)

Trans-disciplinary working

CONTINUITY OF CARE IS CRUCIAL - Jumping from one team to another not good. eg Avoid chopping and changing therapists as much as possible,

Collaborative - no-one "owns" the patient (can still be a culture of this at times)

eg severe aphasia - gone b4 can achieve much

BETTER access to GPs is crucial and NB legally too re decision making/safeguarding etc

Therapy assistance, psychology assistants - in place in Mater ESD; SLTA, OTA, PTA @cuh; Jolene also x3 Assistants (3d pw rehab). KK - HSE/IHF-funded - ? still in place - role - stroke liaison / navigation

Social work support

Home support workers /Case managers

IHF connect service

Each stroke team ideally would have an Aphasia/ emotional support champion

MDT Team, to meet different client needs at different levels, client active in decision making

Mater stroke link - Nurse - point of contact up til outpatient appts - now new role, innitiall S/care

Stroke Strategy - Liaison workers (ward=> home)

SLT OT MSW CPsy/ NPsych at Level 2
All teams members level 1
Level 3/4 Clin Psych /NeuroPsych
PWA ??

The key role of professionals is to disseminate what works to natural supports, who are more likely to be present in sufficient number to make the difference

Zoom allows people with aphasia to come together for groupwork more easily... this is important because aphasia is very isolating

Mentorship

Enhanced funding and lobbying re mentorship from professional bodies

National interdisciplinary supports as there are so many complex intricacies and our national population is small - locally there are mentorship gaps

Opportunity for increased interdisciplinary supervision and mentorship

Community of Practice SEA membership

peer support

Mentorship - ?support from others e.g. aphasia groups, aphasia cafes etc

Having a mentorship programme hub and spoke style for different regions

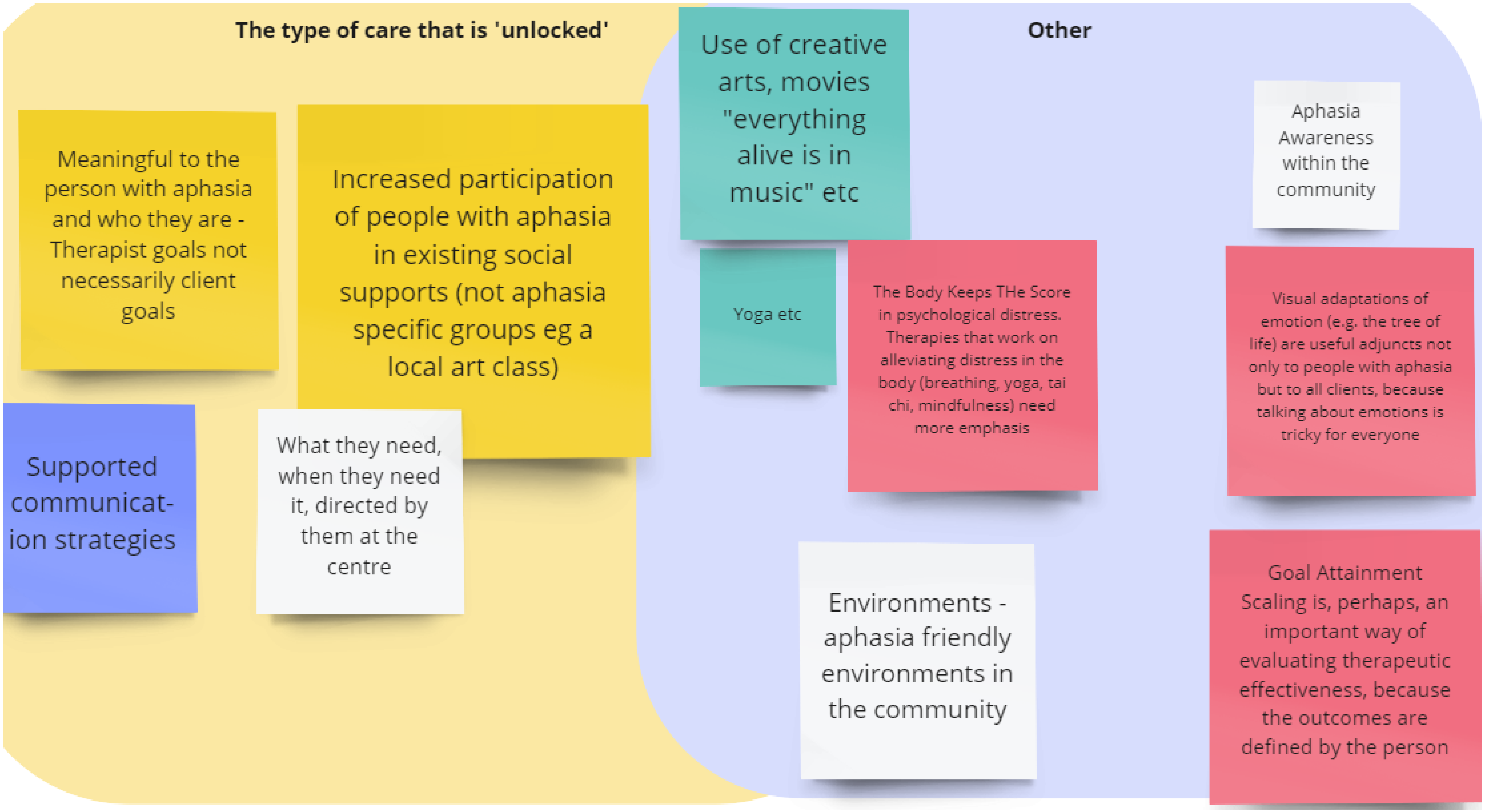
The type of care that is 'unlocked'
Other
Meaningful to the person with aphasia and who they are - Therapist goals not necessarily client goals
Increased participation of people with aphasia in existing social supports (not aphasia specific groups eg a local art class)
Supported communicat-ion strategies
What they need, when they need it, directed by them at the centre
Use of creative arts, movies "everything alive is in music" etc
Yoga etc
The Body Keeps THe Score in psychological distress. Therapies that work on alleviating distress in the body (breathing, yoga, tai chi, mindfulness) need more emphasis
Aphasia Awareness within the community
Visual adaptations of emotion (e.g. the tree of life) are useful adjuncts not only to people with aphasia but to all clients, because talking about emotions is tricky for everyone
Environments - aphasia friendly environments in the community
Goal Attainment Scaling is, perhaps, an important way of evaluating therapeutic effectiveness, because the outcomes are defined by the person

SESSION 3

THURSDAY 2 JUNE 2022

Today

1. Quick recap on sessions 1+2.
2. Aphasia PPI contributor comments.
3. Aphasia Stepped Psychological Care.

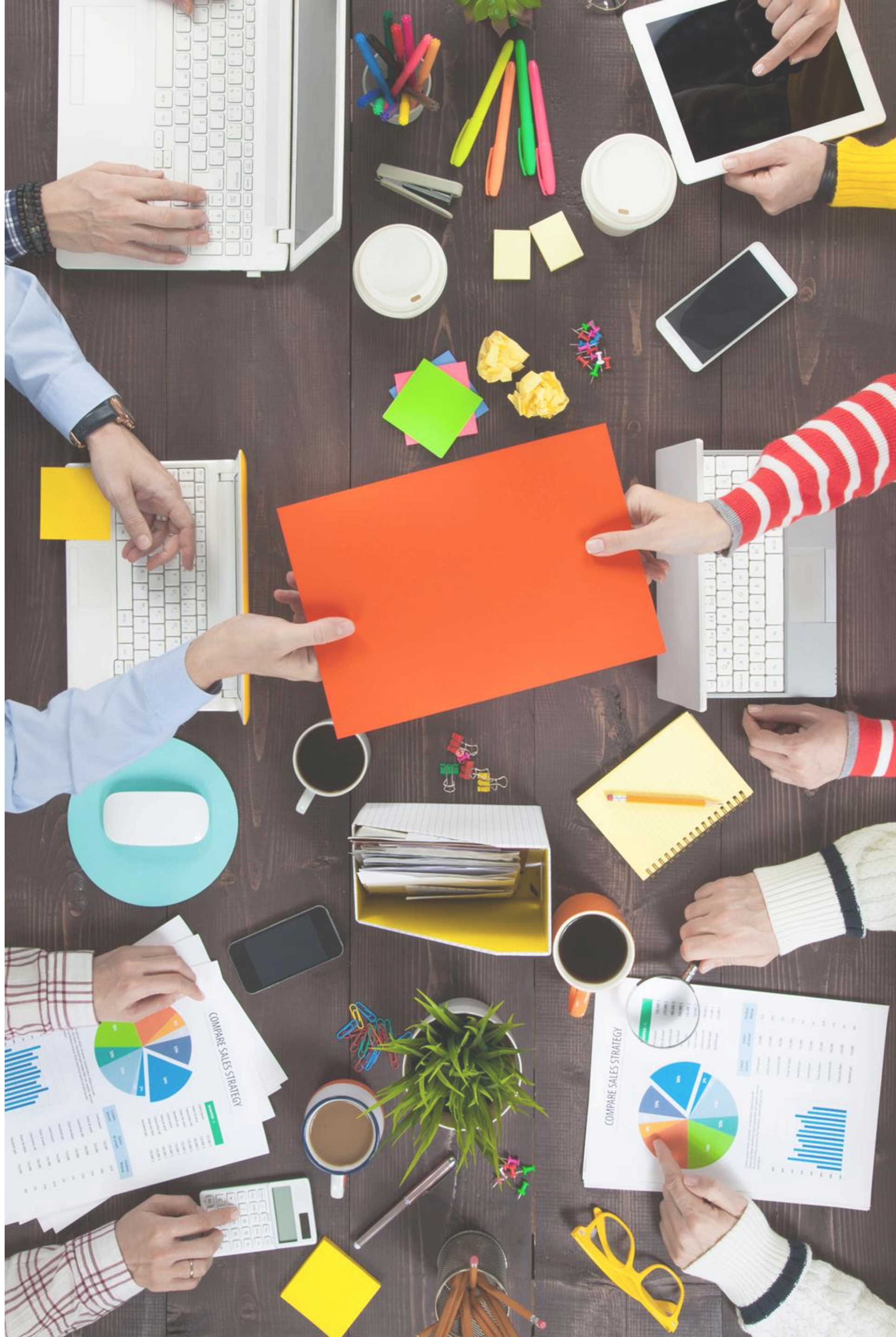

Recap | sessions 1 + 2

✓ SCOPE 13.5.22

✓ ISSUES + CONSIDERATIONS 6.5.22

- ↓ Access + variation
- ↓ HCP understanding of support needs
- ↓ Structural support for HCP
- HCP training
- Different needs @ different stages
- Signposting ≠ the destination

✓ "IDEAL" PRINCIPLES 13.5.22

1) A better pathway

2) Team structures, resourcing

3) Training + support for clinicians

4) Focus of care

✓ PPI feedback 30.5.22

1

1) A better pathway

- Equity, consistency of access.
- Self-referral capacity.
- Care is responsive (needs, timing).
- Continuity of care, relationships.
- Family, couples support.
- Supporting care transitions.
- Liaison support.
- Universal design culture.

Delighted that a group of non-SLTs are interested in emotional support with aphasia - a good mix.

Long-term and self-referral good ideas.

Great idea to be able to go back to clinicians later if you have a problem.

Of all the things that's really great. You can do courses etc., but if you get stuck and want to 'back off' a little, it's a great thing to be able to talk to them about anything - e.g., your speech, your head (mental health).

Acute care | ESD | I/P Rehab | CRT | HSE community | 3rd sector

1

1) A better pathway

- Equity, consistency of access.
- Self-referral capacity.
- Care is responsive (needs, timing).
- Continuity of care, relationships.
- Family, couples support.
- Supporting care transitions.
- Liaison support.
- Universal design culture.

Great idea to see same person each time - just like Dr, knows you, has your files etc.

In 1st year after stroke, always saw same SLT, PT. This team was 'set in stone'. This helped.

Not the way for everyone. Wonder what made the difference (was it that wife is a nurse?)

agrees - knowing people who can help you makes a difference

knew lot of nurses in stroke - some were neighbours - they helped me + husband - people you know will help you

Acute care | ESD | I/P Rehab | CRT | HSE community | 3rd sector

1

1) A better pathway

- Equity, consistency of access.
- Self-referral capacity.
- Care is responsive (needs, timing).
- Continuity of care, relationships.
- Family, couples support.
- Supporting care transitions.
- Liaison support.
- Universal design culture.

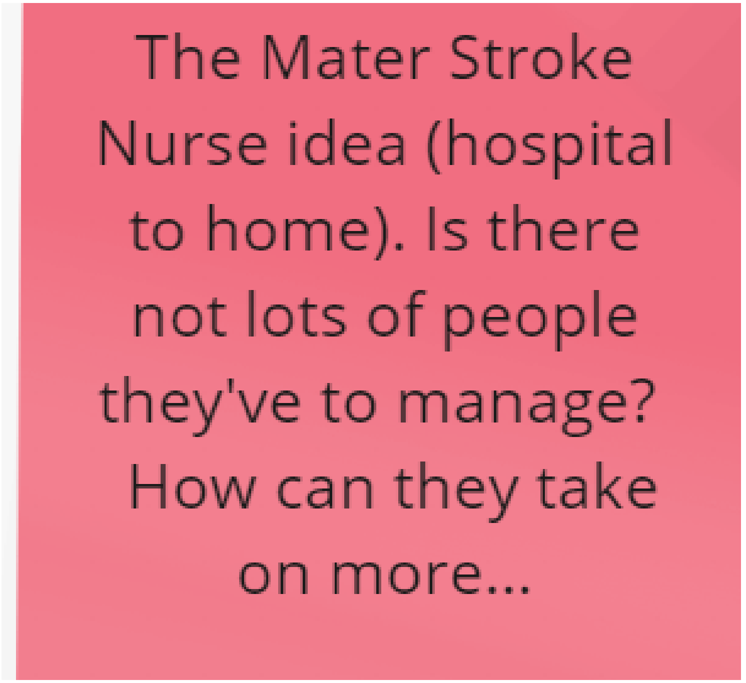

Acute care | ESD | I/P Rehab | CRT | HSE community | 3rd sector

✓ PPI feedback 30.5.22

2

2) Team structures + resourcing

- Better use + coordination of staff.
- Transdisciplinary support (80%).
- Rehab assistants incl. community.
- Protected specialist resource (e.g., ESD).
- But better cost-effectiveness.
- Aphasia ‘emotional support’ champion.
- Home Help beyond personal care.
- Education for policy developers + managers.
- NNB need to capture *valid* clinical, economic, societal outcome data.

clinicians need to work together better.

Less of 1 person doing x, another person doing y - they've to "click" for aphasia

1 person, 1 thing...we need to sort this out

At NRH saw lots of people - SLT, OT etc. but not sure how well they worked together.Clinicians also need to talk to each other.

Who do we mean by the 'aphasia champion' on stroke teams?

3

3) Training + support for clinicians

- Management support locally.
- Mentorship; national interdisciplinary support network.
- Professional body support + lobbying.
- Online training / Interactive workshops.
- Aphasia training for HCP.
- Including knowledge of wider non-medical support needs; supporting people to live well / autonomously.
- Counselling training SLT.

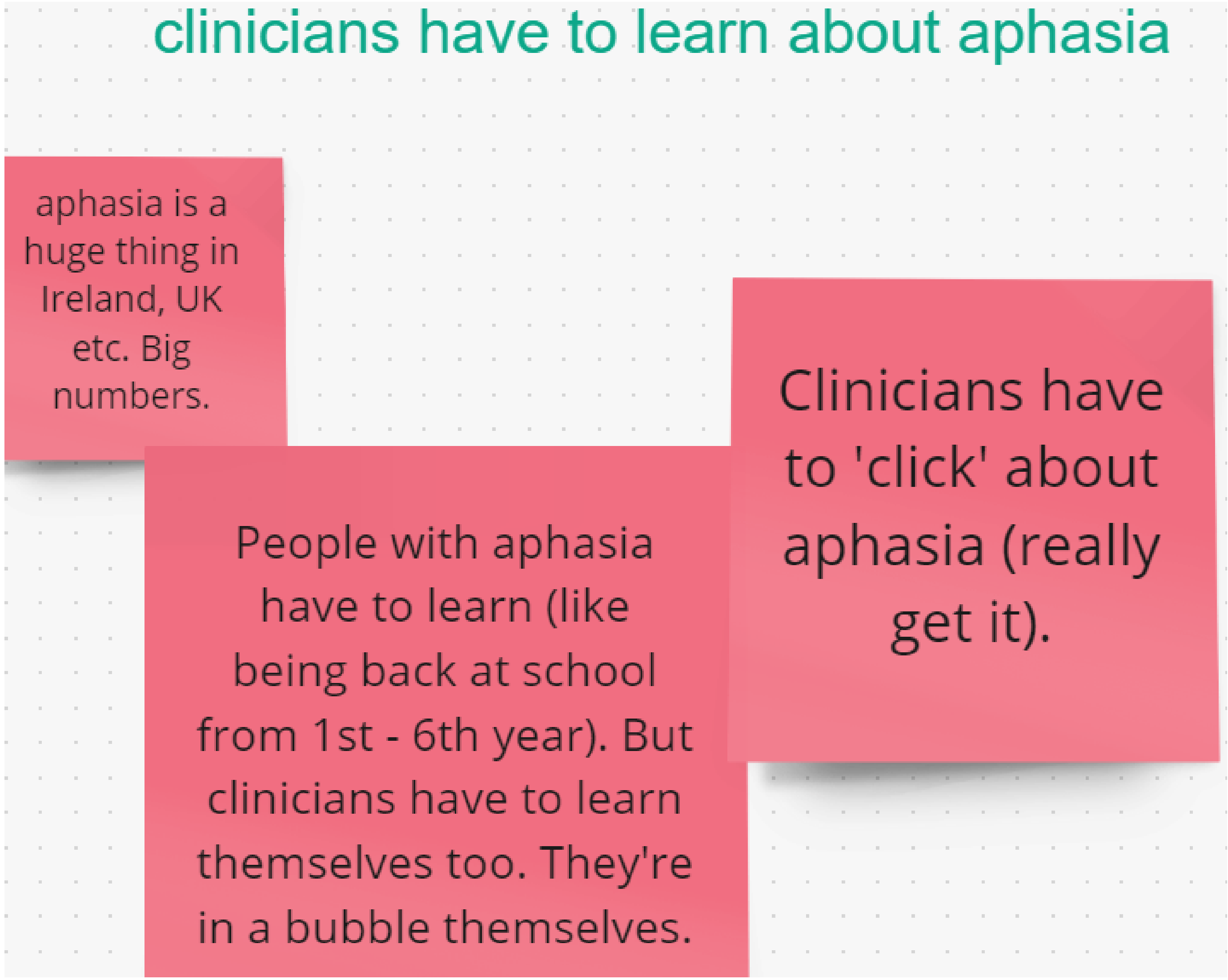

3

3) Training + support for clinicians

- Management support locally.
- Mentorship; national interdisciplinary support network.
- Professional body support + lobbying.
- Online training / Interactive workshops.
- Aphasia training for HCP.
- Including knowledge of wider non-medical support needs; supporting people to live well / autonomously.
- Counselling training SLT.

clinicians not getting it...

NRH OT didn't click about aphasia. Was talking to another person during cooking session (so bread was too wet). Didn't value my deeper knowledge re baking.

Stroke Ward: SHO very rude - talked only to Stroke Consultant ("look at her, her lip is going down here"; dehumanising.

NRH PT didn't listen or help my knee issue / fluid - thought my walking was normal. I wasn't able to explain that there was fluid + it didn't feel ok. So no help given.

felt that people didn't understand why was in NRH - look fine - walked - speaking an invisible problem.

in a little world to yourself (speech difficulty in NRH)

4

4) The focus of care

- Living well.
- Person-centred + integrated care.
- Being part of the community; doing things that interest you.
- Self-care + acceptance.
- (aphasia-friendly) Psychosocial care.
- Behavioural activation.
- Creative therapies.
- Aphasia community + peer support.
- Aphasia-friendly environments.
- Self-management.
- Services not end-point.

person centred care NB

Loves the focus

They need to learn to sit down + listen to the person as well. What can I do to help you?

self-care & acceptance NB - lots of things people can be doing to help themselves (self-management - e.g., swimming etc.)

Need to accept where you are at, then move forward.

have to live life as well - not just services

4

✓ PPI feedback 30.5.22

4) The focus of care

- Living well.
- Person-centred + integrated care.
- Being part of the community; doing things that interest you.
- Self-care + acceptance.
- (aphasia-friendly) Psychosocial care.
- Behavioural activation.
- Creative therapies.
- Aphasia community + peer support.
- Aphasia-friendly environments.
- Self-management.
- Services not end-point.

Clinicians great people -but don't get it.

SEA CoP great, but not thinking of the person - want someone that has same problems at home.

Want people with similar experience to help / peer support.

meeting others living with aphasia / stroke very important

4

4) The focus of care

- Living well.
- Person-centred + integrated care.
- Being part of the community; doing things that interest you.
- Self-care + acceptance.
- (aphasia-friendly) Psychosocial care.
- Behavioural activation.
- Creative therapies.
- Aphasia community + peer support.
- Aphasia-friendly environments.
- Self-management.
- Services not end-point.

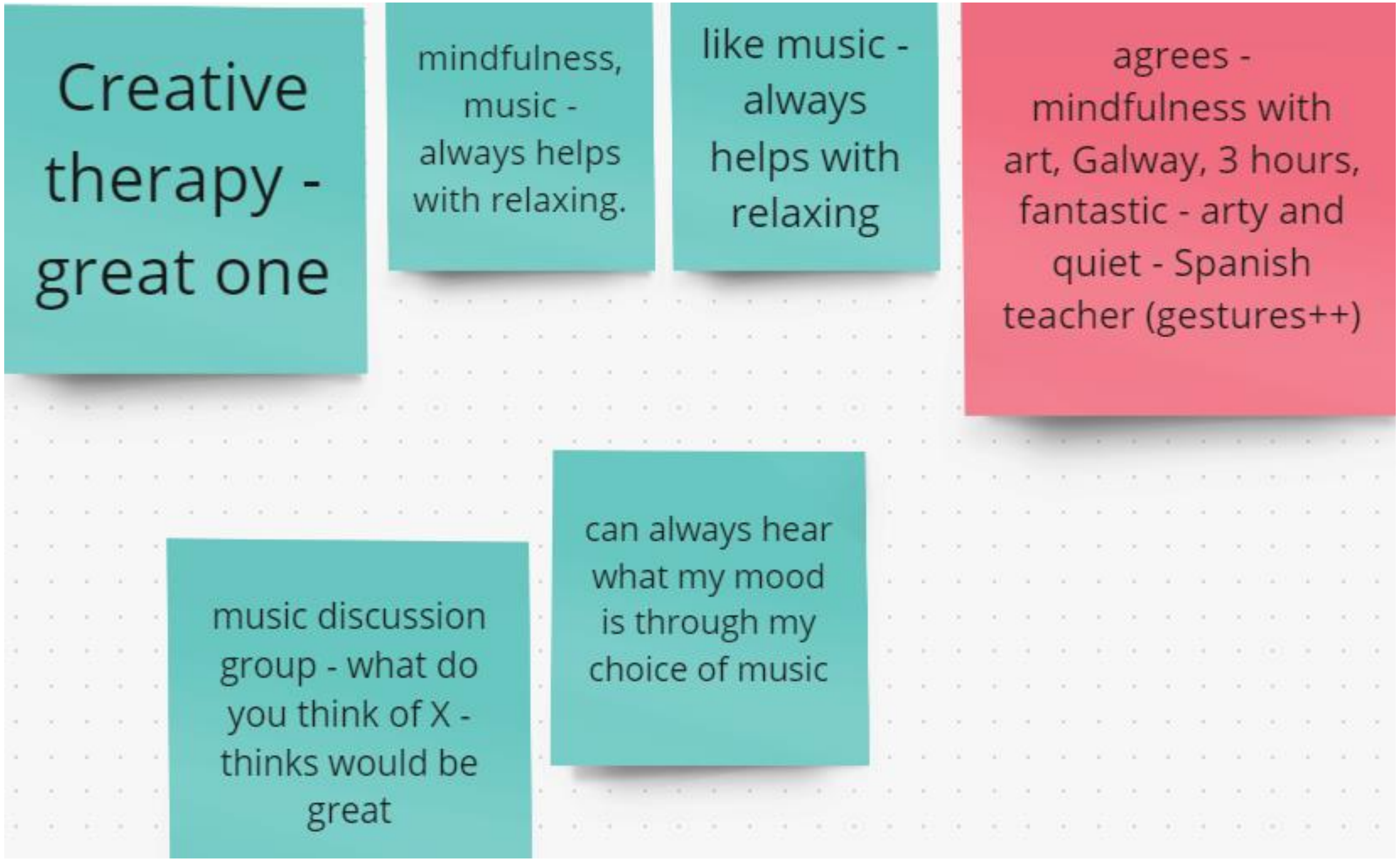

Today

1. Quick recap on sessions 1+2.
2. Aphasia PPI contributor comments.
3. Aphasia Stepped Psychological Care.

Have you any comments?

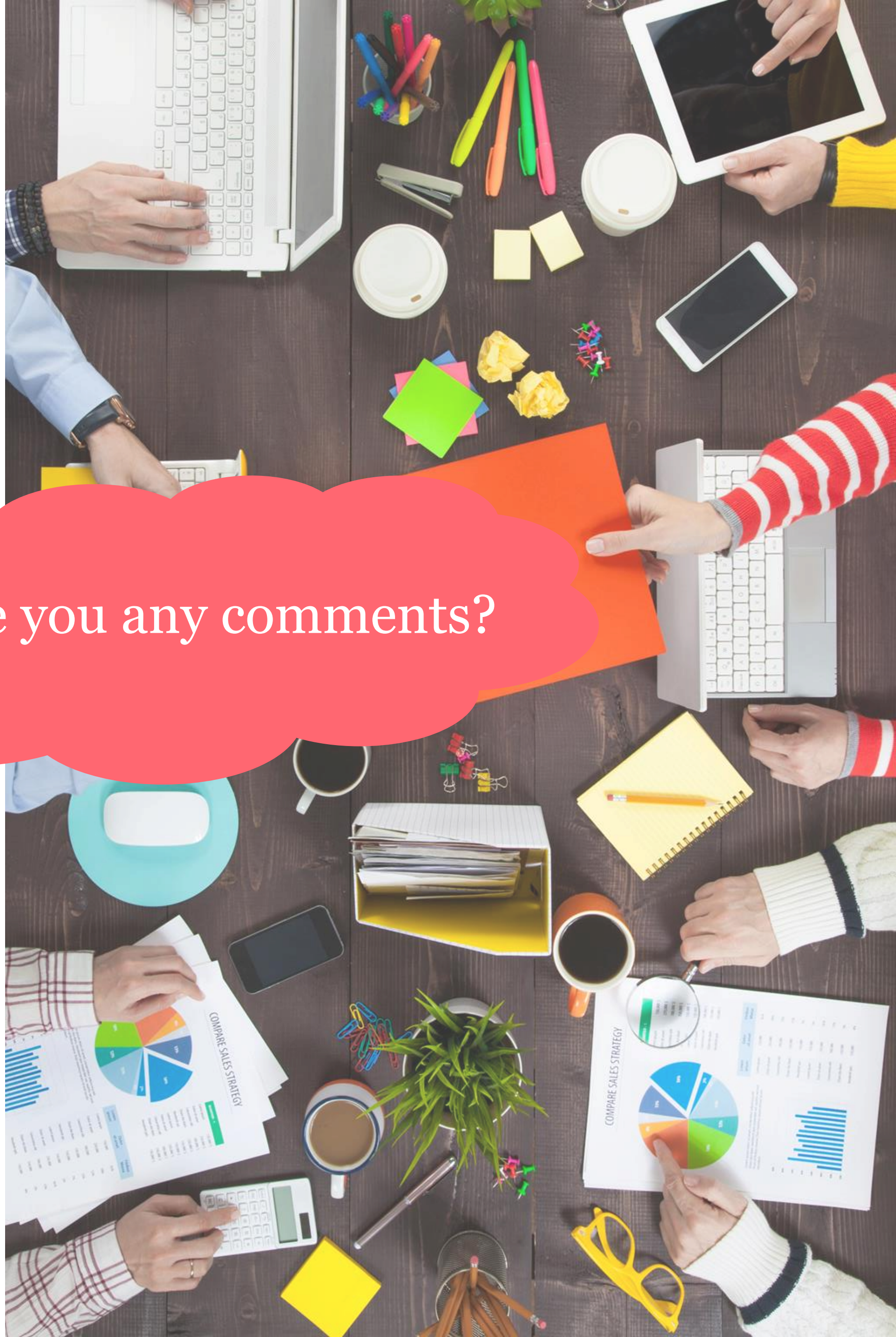

Today

1. Quick recap on sessions 1+2.

2. Aphasia PPI contributor comments.

3. Aphasia Stepped Psychological Care.

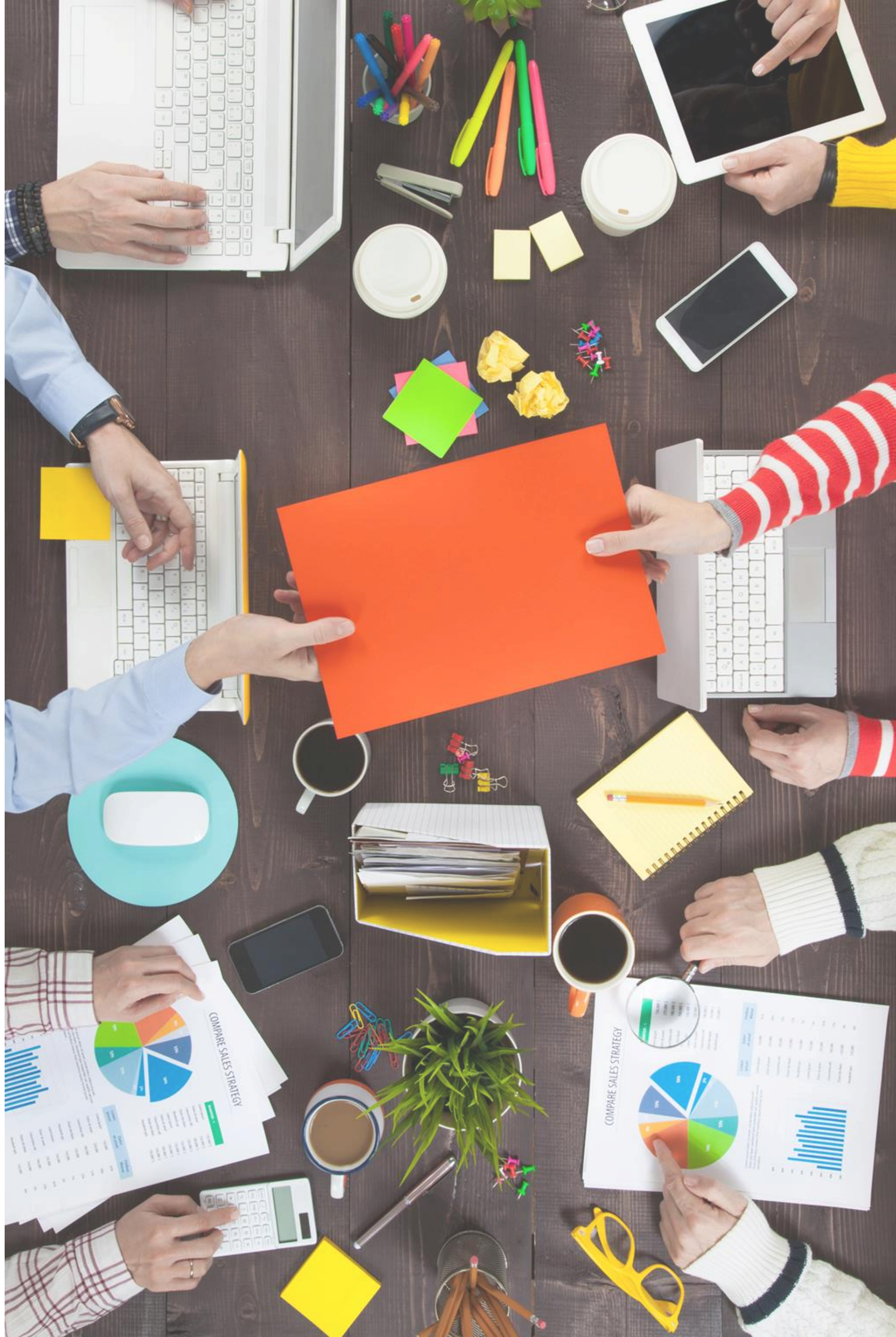

Stepped care model = an **integrated pathway of psychological care** from:

- universal interventions
- initial screening +/- triaging to more specialist support.

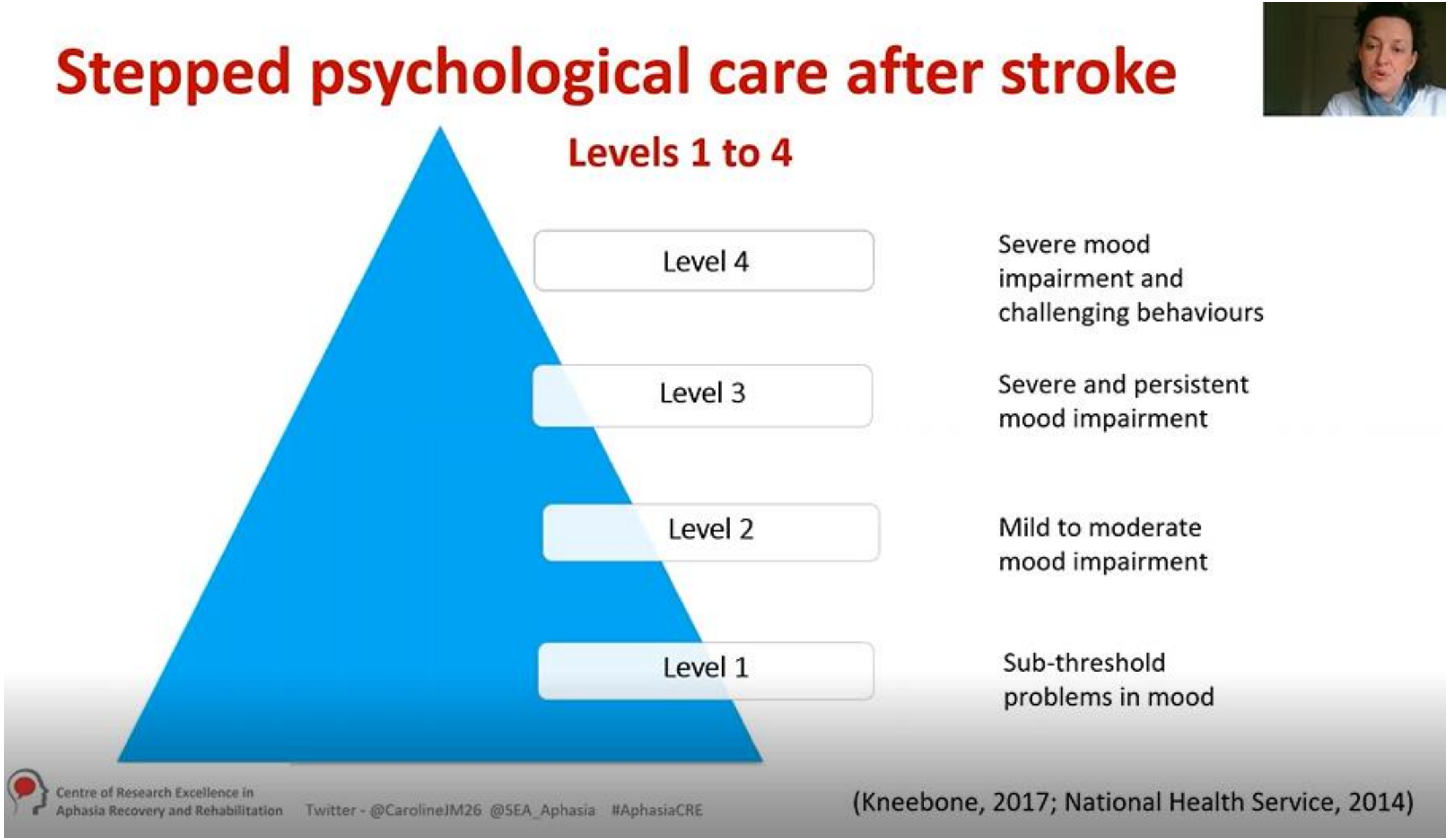

This slide includes a still from Dr Caroline Baker's excellent SEA 2 presentation.

Ongoing research around adapting for aphasia + specifying:

- Therapeutic interventions.
- SLT role.
- Potential for reciprocal interdisciplinary support, mentorship & collaboration.

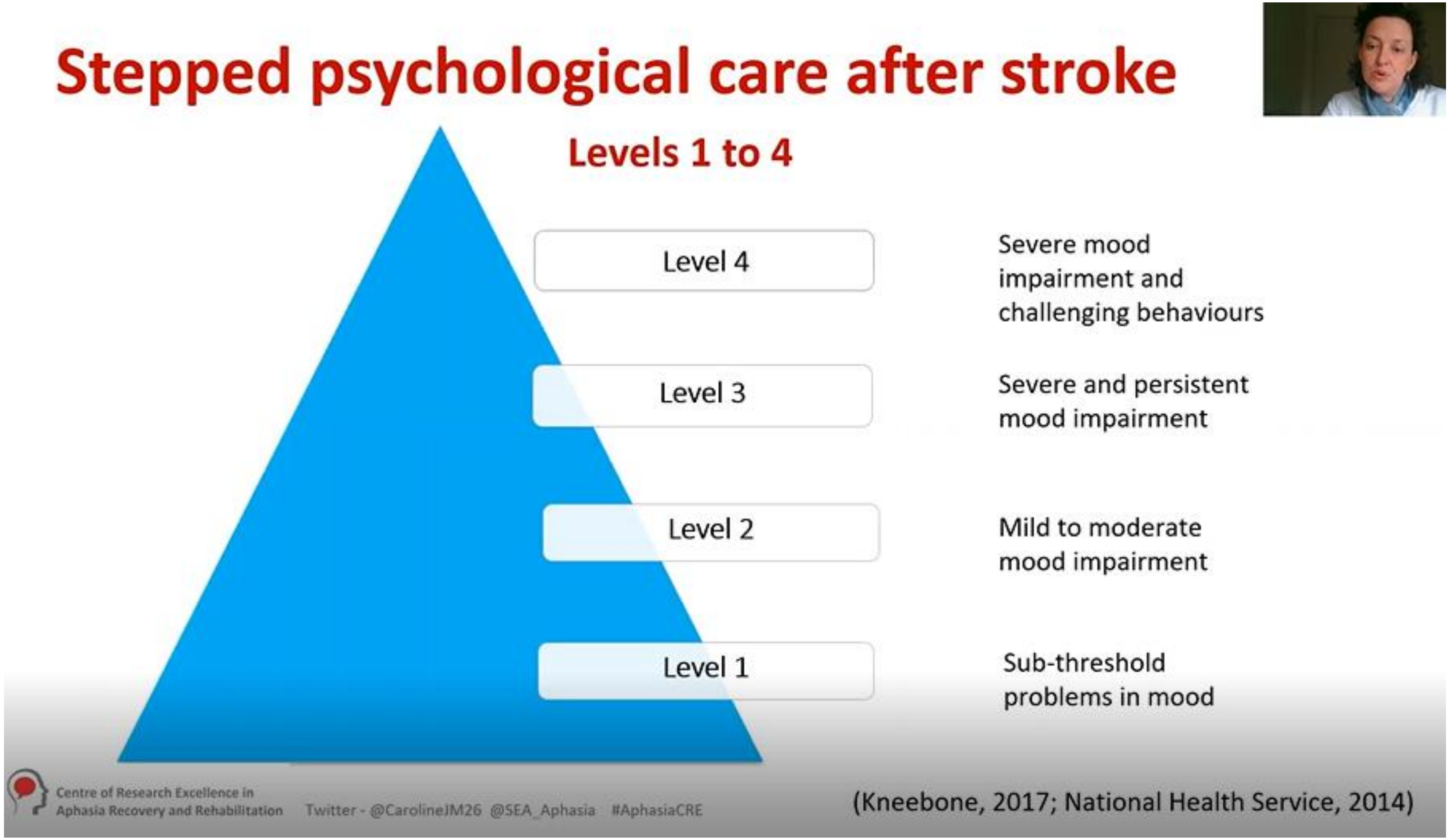

This slide includes a still from Dr Caroline Baker's excellent SEA 2 presentation.

My initial thoughts

“Stepped Emotional Support with Aphasia” Ireland

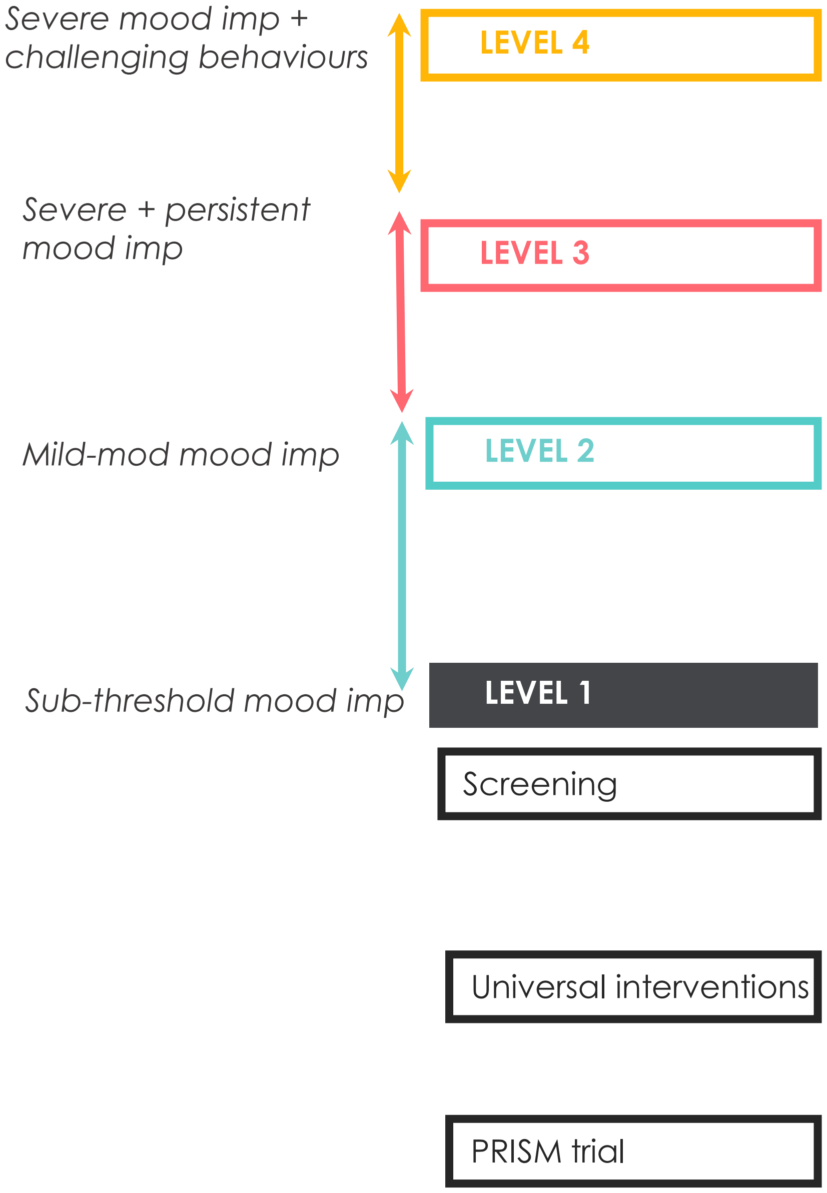

- ✓ Interdisciplinary + integrated model.
- ✓ Operationalises policy.
- ✓ Potential for communicating with managers, professional bodies, funders, policy makers + for dovetailing with other care pathways.
- ✓ Useful for HCP training, mentorship + transdisciplinary working.
- ✓ Can adapt ‘toolkit’ of interventions as new evidence comes on stream.

My initial thoughts

“Stepped Emotional Support with Aphasia” Ireland

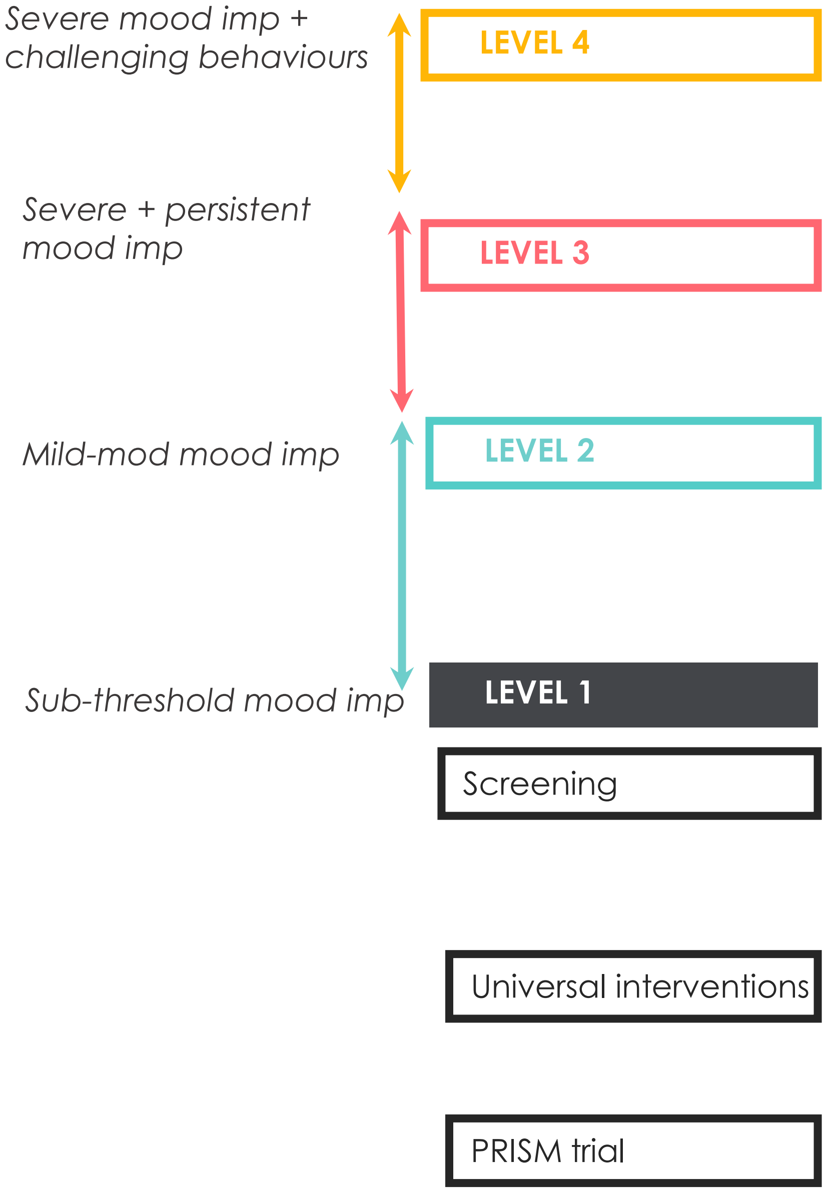

- ✓ Interdisciplinary + integrated model.
- ✓ Operationalises policy.
- ✓ Potential for communicating with managers, professional bodies, funders, policy makers + for dovetailing with other care pathways.
- ✓ Useful for HCP training, mentorship + transdisciplinary working.
- ✓ Can adapt ‘toolkit’ of interventions as new evidence comes on stream.

- How would this work:
  - Longitudinally, across personally-defined recovery?
  - Across continuum of care settings, sectors?
  - How will it be flexible, responsive –What is self-referral access point?
- How would it interface with more general aphasia / stroke /ABI / mental health care pathways? (not ‘ad hoc’)
- To what extent are ‘pieces’ already being done; what are the new bits?

Today

1. Quick recap on sessions 1+2.

2. Aphasia PPI contributor comments.

3. Aphasia Stepped Psychological Care.

- ✓ Is it useful?
- ✓ Does it cover our scope?
- ✓ (How) might it work in our context?

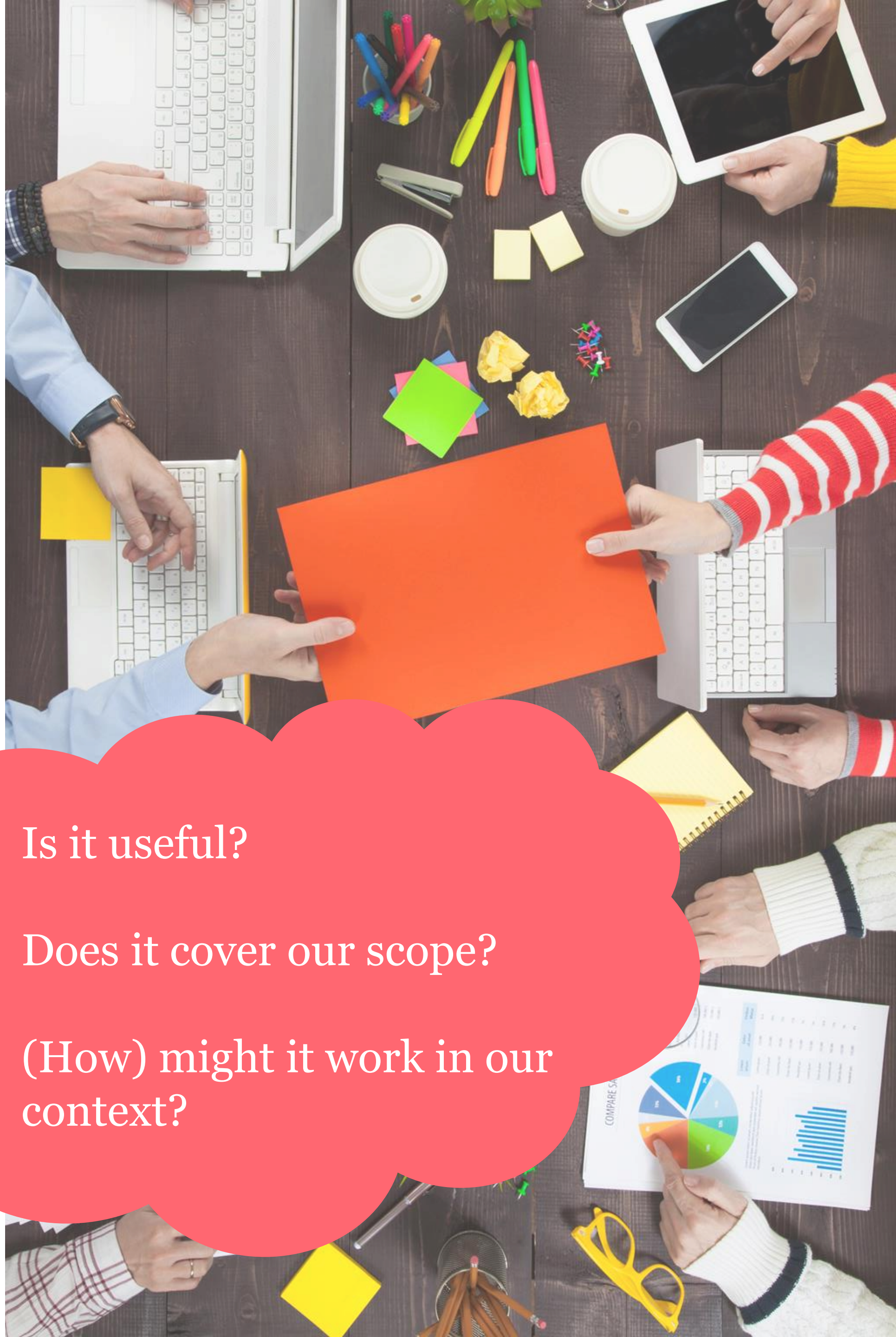

- ✓ Is it useful?
- ✓ Does it cover our scope?
- ✓ (How) might it work in our context?

What is the value of aphasia stepped care?

- (How) might it work in Ireland? Is it feasible?
- What is already being done versus new bits?
- What might be levers / supports for implementing?
- How could it interface with other pathways?
- What is priority?

jolene marcia brian libby aoife

SESSION 4
FRI 10 JUNE 22

Today

Any thoughts / comments?

1. **Re-visit your Aphasia SPC reflections.**

2. What should be prioritised / where to start?

3. Stakeholders & Dissemination

4. Close out sessions.

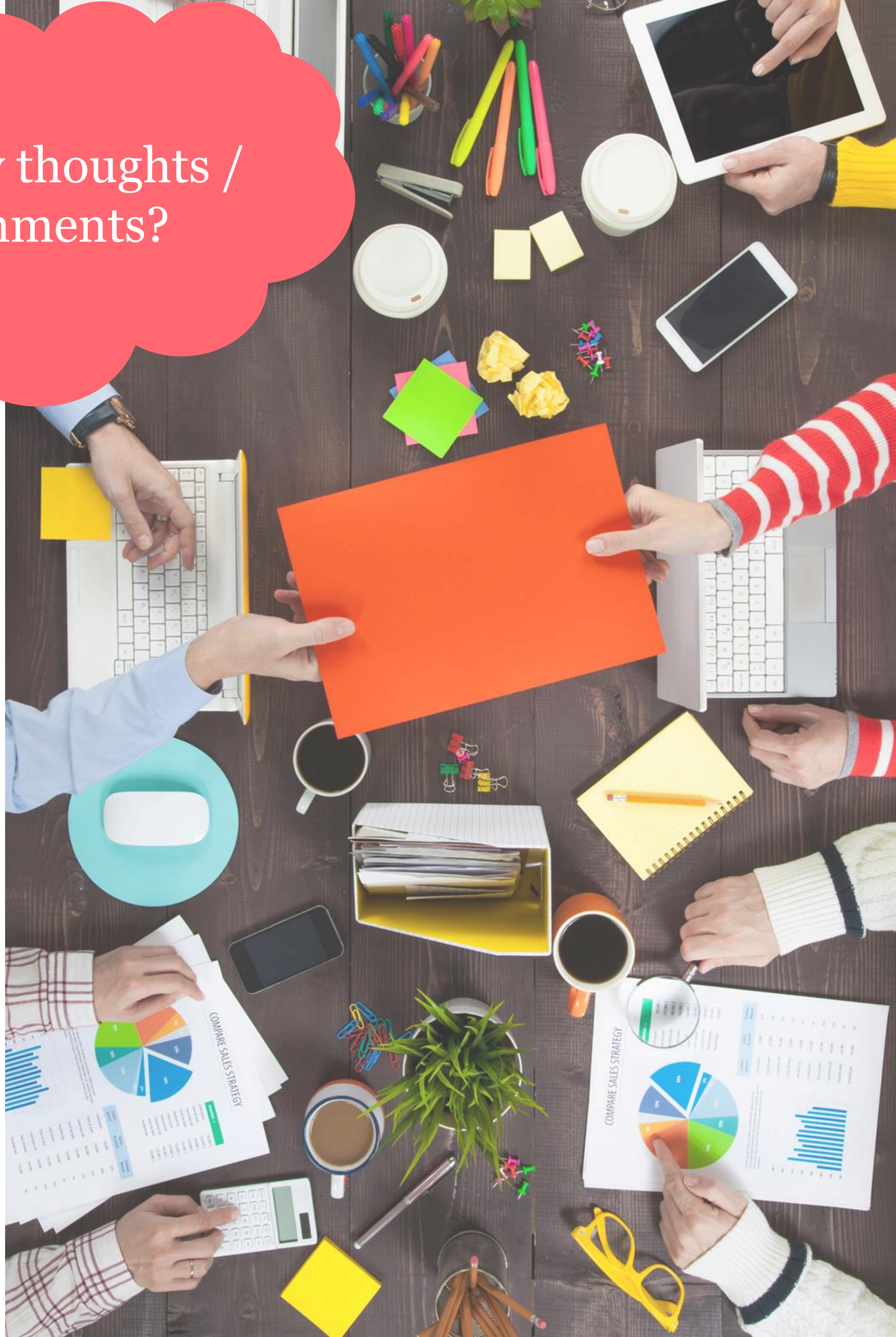

What are potential implementation levers?

- **New ECCNs** (primary care network managers)
- **SEA CoP** (brings many opportunities for interdisciplinary learning & integration)
- Existing L1 **IHF supports**
- Exploring the **feasibility of establishing links with IHF stroke support networks** with stroke services to emulate the benefits of peer support as Jolene's story demonstrated
- "Results from this **co design research should be used to inform stroke policy** and tie in with drafting of the **HSE Psychological Guidance** document"
- "Need to link in with **clinical leads & present at medical conferences** etc re publicising need & introducing model"
- Need for recognition that SLTS provide amazing emotional, compassionate care to their patients ,so sometimes its more **reassurance that we are not missing something and confidence in existing skills and practice**

Need for recognition that SLTS provide amazing emotional, compassionate care to their patients ,so sometimes its more reassurance that we are not missing something and confidence in existing skills and practice

There already are some Level 1 supports eg through IHF

Results from this co design research should be used to inform stroke policy and tie in with drafting of the HSE Psycjhological Guidance document

Need to link in with clinical leads & present at medical conferences etc re publicising need & introducing model

Exploring the feasibility of establishing links with IHF stroke support networks with stroke services to emulate the benefits of peer support as Jolene's story demonstrated

What are potential implementation levers?

I have had more meetings and information from the Psychology services nationally and internationally and Psychologists since we started the SEA CoP. It has been an eye opener. It IS possible for us to integrate our work!!

I think the new ECCNs might help eg no more Discipline specific line managers ie a primary care network manager for all, an ICPOP manager etc therefore promoting a team approach - I do think having different line managers/pots of funding leads to an "us" versus "them" unidisciplinary wotking style eg that service has more staff than us therefore we can't reach on this new initiative until we get what they have etc

What are potential barriers to implementing change?

- SLTs **'firefighting'** – little room for service development / new initiatives, running support groups – individually managing wait lists and staffing challenges.
- **Lack of clarity** around **what's already happening around country** (and **variation**).
- Attitudes / culture: "**Not to be caught up in the "that doesn't exist** or the approach as then nothing will change"
- **"Alternative modalities" / Creative therapies (or non-orthodox) are often seen as a luxury item and peripheral, rather than core** services. Aphasia should turn this perception inside out... if I cannot talk, access to **other forms of expression is essential**, and **talk therapy is peripheral**.
- Aphasia sometimes 'missed' by SLT when dysphagia screening / physical impairment prioritised.

What is being done already? it like varies throughout the country

I believe LEVEL 1 COGNITIVE SCREENS are needed too. AND language screening for everyone post ABI and Stroke as we still receive plenty of refs in community for people whose language was never screened - APHASIA has been missed by SLT colleagues who screen only for dysphagia at hospital level due to staffing demands and lack of prioritisaiton re: language/cognitive/emotional vs "physical" impairments. I think the interventions need to be interlinked and a tea approach is the only way to achieve this

"Creative therapy" – put to side – same for holistic and non orthodox. Like trauma work / PTSD, "talk therapies" don't always help . Body keeps score – "alternatives to talk therapy" rather than language of "creative".

Not to be caught up in the "that doesnt exist or the but,but, but approach as then nothing will change

What are potential barriers to implementing change?

Creative therapies are often seen as a luxury item and peripheral, rather than core services. Aphasia should turn this perception inside out... if I cannot talk, access to other forms of expression is essential, and talk therapy is peripheral

We try to run support groups but they are time consuming to organise and in the context of bloated waiting lists and staffing challenges we are inclined to return to individual "fire fighting" rather than service development/new initiatives

Person-centred and integrated care:

1. **MDT/Transdisciplinary working** is NB in all settings, all levels of care
2. Duration of interventions should be "**as needed**" rather than "8 weeks" as this is "what we are funded for" etc
3. **CONTINUITY** IS NB NB NB RE RAPPORT BUILDING AND PERSON CENTRED CARE.
4. Need for acknowledgement that **quality of conversation changes** for people to be more passive and more instrumental /care based.
5. Real need for initiatives where people can feel **joy and purpose**.
6. **Normalising** (v pathologising) and **validating** distress in the context of aphasia
7. Needs to be **fluid** across the stroke care continuum
8. Interventions are more effective when recipients can **choose from intervention menus**
9. re yoga etc, a schedule of classes that people can book **on ad hoc basis/ meet up with peers**
10. A **change in culture** to ensure that **time is made for the psychological needs** of a stroke survivor.
11. Some touch-based intervention problematic considering 1 in 4 sexual abuse survivors.
12. Consider **Psychological Therapy after BI** model.
13. Care / goals need to be **values-based**.
14. Need **Level 0** – recognition that people bring all experiences to stroke, aphasia.
15. Prioritise personally meaningful goals; recognise **prior life participation** / activities.
16. Need to **change 'shape' of model – e.g., like Personal Recovery model**, non linear; incorporates when people do not receive services.

person-centred + integrated care

Need Level 0: people bring all experiences to stroke + aphasia.

Barbara / Pamela Klonaff - psychotherapy after BI - book, will fw = model of psychological rehab - comprehensive functional model - maps. Better shape than SPC.

Normalising (v pathologising) and validating distress in the context of aphasia

re yoga etc, a schedule of classes that people can book on ad hoc basis/ meet up with peers

Full MDT at all levels of care NB

Care, goals need to be values-based.

Interventions are more effective when recipients can choose from intervention menus

Need for acknowledgement that quality of conversation changes for people to be more passive and more instrumental /care based. Real need for initiaves where people can feel joy and purpose, I

Duration of interventions should be "as needed" rather than "8 weeks" as this is "what we are funded for" etc CONTINUITY IS NB NB NB RE RAPPORT BUILDING AND PERSON CENTRED CARE.

a change in culture to ensure that time is made for the psycholiogical needs of a stroke survivor

Touch - procedural vs therapeutic - problematic in hospital setting - lots of touch interventions: 1 in 4 sexually abused.

Recognise previous leisure participation - focus on participation that is purposeful, meaningful.

Need to change 'shape' - e.g., like Personal Recovery model.

Families

Opportunity for partners of PWA to help others; groups NB.

Family peer support NNB. e.g., of groups in Headway (older may 'adopt', support younger women - informal support in lieu of formal + sense of betraying loved one).

Families should be supported in parallel rather than at a specific level; a continuum of care, embedded in aphasia support.

Family members also - do they have access to support & emotional care. It is likely done unidisciplinary - but PWA tends to be focus)e.g. Rehab setting)

"Family days" should not be about psychosocial eduaction re BI - might be only day 'off'! Massage, warm towel shave etc. Meet others.

"Carer" language is problematic.

Support for family NB also. Needs to be a focus also.

Potential for independent psychological support for family members as required.

As above, continuum, embedded - may have inverse support requirements in longer-term.

Previously co-developed comic with (adult) children of PWA who were <10 at onset - what they'd have liked to know.

Families:

1. Support embedded in parallel on continuum as part of aphasia care.
2. May have support needs that increase over time.
3. Family peer support, groups NNB.
4. Children support (e.g., comic).
5. ‘carer’ language is problematic.

Who do we need?

1. **Advanced HSCP practitioners** in clinical practice …"who can roll out stepped Psychological model of care. I don't think this is a skill that new grad SLTs might have for example."
2. Services should invest in **case managers** that broker services, following through from hospital to home.
3. There needs to be a **case coordinator** overseeing the entire model of care I think.

Involving clinicians and people living with aphasia

1. Identifying a **PWA within a stroke network** that could be involved in educating stroke MDTs on the needs of this stroke population
2. **Family members and frontline staff** can effectively be taught to design multi-element intervention (i.e. for the really "complex" presentations, and multi-element behaviour support research backs this up.
3. Ensuring there is **adequate PPI representation in policy making** e.g. IHF, National Stroke Programme working groups

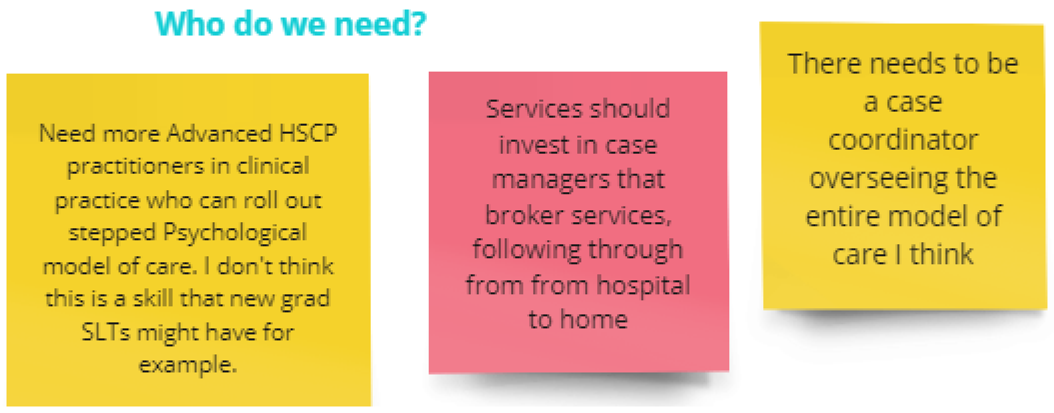

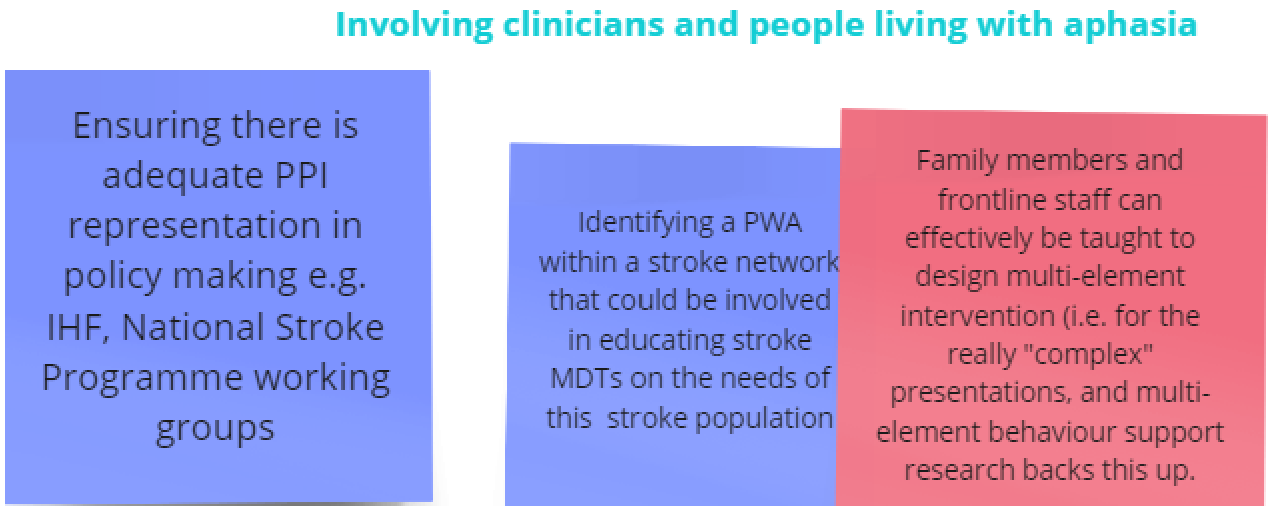

Level 1: Universal

1. Need to **broaden scope**.
2. **Triaging may be problematic** and arise because level 1 under-resourced.
3. **Transdiagnostic groups** are important for living well.
4. Incorporate **"alternative modality" interventions** at all stages etc.
5. Incorporate groups with **peer support** from people who have lived with aphasia loner.
6. Include **cognitive, language + mood screening**.
7. **Community interventions** e.g., home café.
8. Aphasia friendly **communication (health + community) environments**.
9. Aphasia **awareness**.
10. **Early (acute) NeuroPsych, SLT psychoeducation** groups.

Level 1: Universal

Aphasia cafes etc re community long term support

Aphasia friendly environments both in HSE settings & in community - Make Aphasia visible!

Slightly against the idea of levels of intervention, groups often work really well when there are guides who have already travelled the path.

I think the idea of triaging is because there are not enough professionals, but the non-professional, non-"talking therapy" interventions may be more effective, especially thoseinvolving Communication Partners

They're piloting groups on ward (Anne Barrett, Marcia + Amy, SLTA) – SLT & NeuroPsychology for severe aphasia that they'll suspect will be chronic. CPT + psychosocial education intervention – manage distress.

Transdiagnostic approaches NNB for living well.

"Alternative Modalities"

Creative therapies as part of HSE - Would be brilliant in all stages and all environments e.g. Rehab Unit, Community etc

"Alternative Modalities" like Mindfulness, yoga, theatre, biofeedback – really intriguing

I believe cognitive screening is also extremely essential. I think cognitive change post stroke is really inter-related with emotional impact. EG post stroke a persons entire coping style may change, they may not be able to problem solve or sequence as they did before, their attention may be impaired and FATIGUE can be huge. This is not to mention a potential APHASIA on top of this. These areas are all inter-related.

I wonder should non Psychologist HSCPs like OTs and SLTs receive training in screening re mood at Level 1 and also support from Psychology re self care professionally - this puts a lot of emphasis on need for a lot more Psychology and Neuropsychology manpower though and I fear therefore is unrealistic maybe.

As an SLT I ask every client I see post Stroke/ABI how is your mood (in an accessible way) at least and also we use rating scales like AIQ, emotional impact questionnaires, S24 (for those for whom it's accessible) Tree of life/Blob tree, VASES.

Need to broaden / widen scope of L1.

Level 2-4

1. Some elements unclear – e.g., difference between L2-3; when to refer to Psych at L3-4.
2. Limited evidence re Level 4 interventions – not commonly implemented.
3. L4 is multi-element. Umbrella term = MEBS.
4. SLT input NB at higher levels; not commonly implemented.

level 2 - 4

I am not sure about the difference between level 2 and level 3, and whether it is helpful to disntinguish between them

Level 4 interventions are multi-element: adapting environments, particularly adapting teh communicative environment, teaching alternative communication skills focused interventions and reactive plans

I think the umbrella term for Level 4 work is Multi-element Behaviour Support (MEBS)

Guidance on when/who to refer to Psychology services for level 3 and 4

Some evidence for ACT & Aphasia - level 4

There is no evidence for level 4 cause no one is doing it

Level 3 & 4 - specialist care. SLT role support communication to enable PWA to access service - Does this happen?

Level 4 work starts with Functional assessment...a multi-contextual understanding of the meaning or message of behaviour. Behaviour has meaning in a cognitive context, in a communicative context, a life story context, environment context, health anc medical context, motivational context, historical context and situational context

Level 4: SLT NB for communicaiton support. If in MH/PoLL services - SLT not always a member of team, no dedicated SLT in PLL/MH in a lot of areas.

Support and training for clinicians

1. Need **access to supervision for everyone in the team** who is having or supporting emotional conversations is essential. Professional appropriate supervision for SLTs w an emotional support focus is crucial so we can engage in **professional self care** and **problem solve/debrief** re emotionally charged professional relationships/interactions which are very common
2. Training in **psychological strategies** for level 2 for **non-psychology staff** e.g. MI, CBT
3. **Supported communication** education for **all settings** e.g. GP, CNUs, PLL, MH, N/Hs - to make access to services easier - early referral etc. Training for all psychology staff in supported communication
4. Increasing awareness within the MDT of the **need for SLTs to be involved in mood screening**/assessments/discussions re mood with the psychologist and where applicable (OT)
5. **Empathic and active listening skills** – all professionals
6. We (SLT) need to be doing more **joint sessions** with Psychology and OT.
7. As SLTs we need to become more **comfortable letting people "be"** and not always focusing on the "fix“.

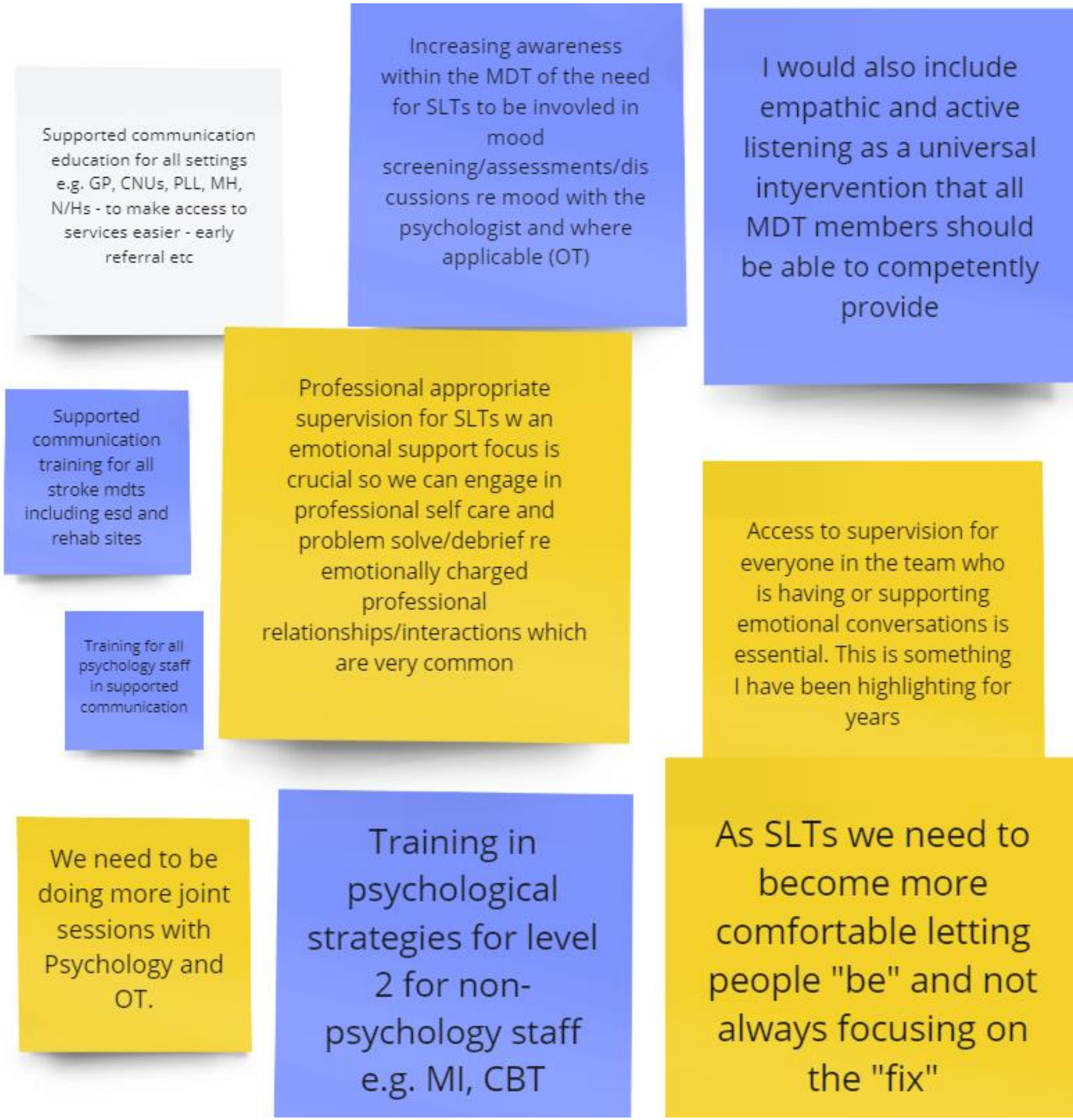

Transdisciplinary working

1. **Transdisciplinary working a pre-requisite**.

Trans-disciplinary team work would facilitate this

Needs to be fluid across the stroke care continuum

A stepped psychological model would be SO useful but we need functional, integrated teamwork to achieve this

MDT/Transdisciplinary working is NB in all settings to achieve this

Ward has SLTA, OTA, PTA

Today

1. Re-visit your Aphasia SPC reflections.

2. **What should be prioritised / where to start?**

3. Stakeholders & Dissemination

4. Close out sessions.

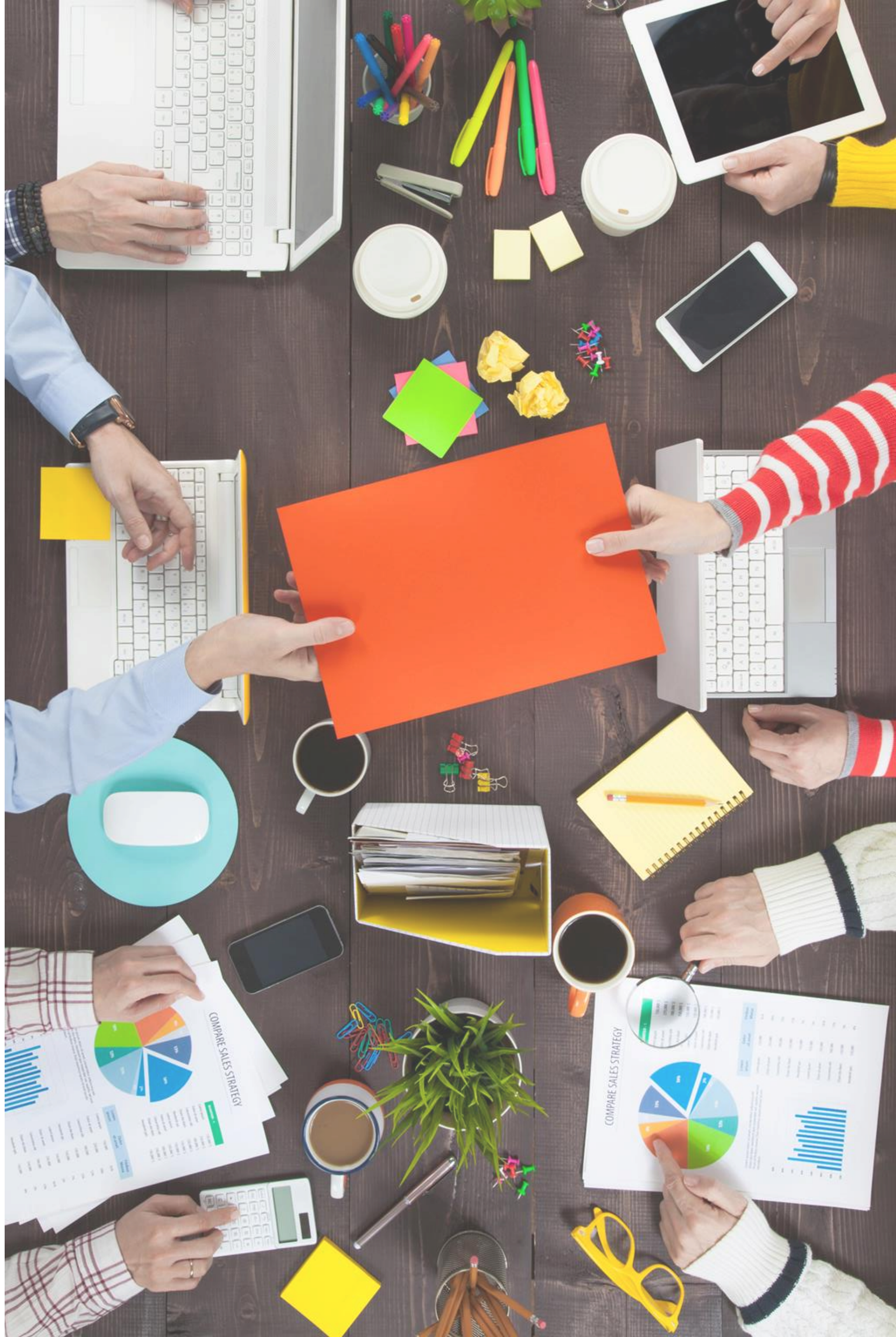

What are priorities / where to start?

Adapting health communication environment

Service design

Dissemination & impacting policy

Family support

Self-management & Peer support

"Alternative modalities"

Clinician knowledge & support

- Aphasia awareness across continuum of care
- Clinician support & self-care
- Self-directed / online
- Pre-qualification training
- Interdisciplinary training / co-learning

What are priorities / where to start?

Pre-Post communication training one ward / one unit / IHF etc.

Adapting health communication environment

'Communication accessible' hospital - plus "Award" - embedded in HSE Health Literacy / Access Policy etc.

Thinking about adapting environments to create a greater fit with people's needs and characteristics, instead of individualising problems in communication

What are priorities / where to start?

Service design

mapping what's already being done

'joining dots' on stroke liaison role across policies / practice

further co-design sessions to develop 'shape' of model - wider stakeholder consultations

Idenitfy PPI to be involved in design and roll out of a national model e.g. PWA champion attached to a stroke network

PPI / co-design 'alternative' modality intervention suite

Dissemination & impacting policy

publish this research and get it on the desks of the key strategy leaders

Link w our professional bodies and insist that this is needed ASAP - the current model just isnt fit for purpose and it's completely haphazard !

Position paper endorsed by HSCP Organisations

PSI/DON Event /older adult services

Go to the media?? Although many of us are "gagged"

Get the gravity and seriousness of this need front and centre w the medics and Heads of service. How can we all "talk the same language" and see the sheer importance of this in terms of living well and yet every day we have to fight and argue as clinicians to keep our clients i[illegible] services/ on a rehab ward...

Irish Gerontological Society Conference / Stroke conferences

...I see these supports as "what makes life worth living" but the Irish system is obsessed with PHYSICAL outcomes eg OTs in community only have capacity to deliver equipment not able to do all of their wonderful and necessary cognitive and psychological assessments and interventions

What are priorities / where to start?

Family support

communication is never solo, so family based interventions should be prioritised.

getting the cartoon / comic rolled out! even in different format - POWTOON etc.

"Alternative modalities"

Review the efficacy of alternative modality therapies to enhance emotional wellbeing: biofeedback, neural feedback, yoga, tai chi, massage, mindfulness, theatre,

lINKING IN WITH UNI COURSES DRAMA/ MUSIC THERAPY ETC RE PROVIDING SERVICES

Dissemination before therapies. What would a training course in emotional support for people with aphasia look like?

begin with the outcome in mind, living well with aphasia is not a post therapy idea

consider aphasia ambassadors, people who are already living well with aphasia who can signpost newbies

Self-management & peer support

looking at existing SM / psychosocial / training interventions + seeing how to adapt

specific PRISM intervention (acute / post-acute, 1st 6 mo) pilot 1 health area

What are priorities / where to start?

Clinician knowledge & support

Awareness in all areas - Acute - Rehab - Community etc - can be done through education sessions. Include PWA in this - they are the experts!

Aphasia awareness across continuum of care

People with aphasia are in all environments e.g nursing homes, PoLL units, as well as at home - we cant forget these environments. Access to AHPs in Nursing Homes is very hit and miss.

Make Aphasia Visible - in all environments. Make it a priority in all environments e.g. for SLT, huge number of referrals for dysphagia & difficulty with language may not be mentioned on referral!

Clinician support & self-care

clinical supervision / emotional support should be mandatory for all professionals. How can someone give emotional support if they don't experience it?

Self-directed / online

Explore Future Learn, Udame, eLearning Life

Online self-directed supported communication foundation on HSELand...like Aphasia Institute's one.

? Online STARS training

What are priorities / where to start?

Clinician knowledge & support

Pre-qualification training

More interdisciplinary workshopping at undergrad training level re complex cases like this

GPs sould have to spend a week with each HSCP discipline as part of their training - the AWARENESS IS NOT THERE

More structured education re Psychology, OT and SLT for medics and nursing staff at Undergraduate level

Design series of Education sessions for stroke MDTs including topics such as supported communication, how to support the PWA on their journey to adjustment (include here about not the aim of rehab NOT to be "to get back to normal",

Topics for stroke mdt sessions continued, acknowledge the stroke occurrence and impact of same, reassurance, outline aim of rehab is to drive neuroplasticity, change is possible whilst gentle encouragement to not "get back to normal"

Interdisciplinary training / co-learning

accredited aphasia support training course delivered by different disciplines

Today

1. Re-visit your Aphasia SPC reflections.
2. What should be prioritised / where to start?
3. **Stakeholders & Dissemination**
4. Close out sessions.

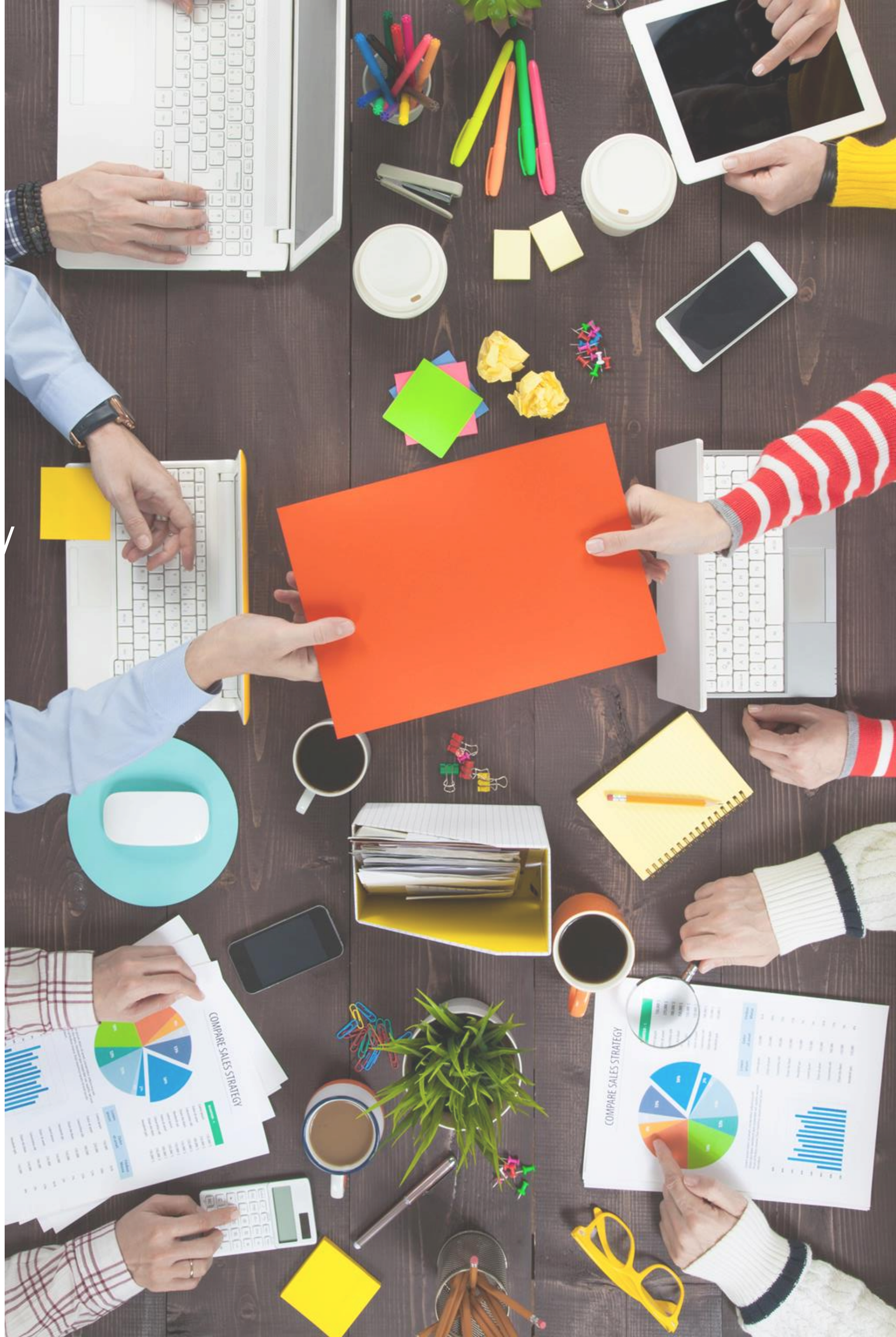

Who are the stakeholders?

- ? Policymakers
  - Hse, DoH&C, icpop, ECCNs, managers, professional bodies, 3rd sector support orgs incl. IHF, ABII etc., NAI, Clinical Leads, Neurorehab policy, HSE Psychological Guidance, Professional bodies, HSCP Office…
- Clinicians, Consultants, GPs, Stroke Nurse Specialists
- SEA CoP
- Wider HSE / site staff
- Patients / Caregivers / Aphasia advocate networks
- Ehealth
- HSE Communications
- Quality & Patient Safety

Stakeholders | 10.06.2022

POLICY

IHF / Council on Stroke

ABII

nai

Sláintecare

stroke programme

noca

IASLT/AOTI / ISCP

Other prof bodies

Neuro-rehab strategy

EXISTING NETWORKS

SEA CoP

international aphasiologists

Irish aphasia researchers / SLT courses

colleagues' professional networks / SIGs

SPHeRE network

Stroke Cog

ARC research centre

Stakeholders | 10.06.2022

- HSE/DoHC
- eHealth
- HSE Comms
- quality & patient safety
- ECNN managers
- ICPOP
- stroke units
- ESD services
- nrh
- health literacy
- HIQA
- Inpatient rehab sites
- Clonskeagh Stroke Community Team
- ED staff
- DayCare staff

- CLINICIANS / STAFF
- OT SLT PSY
- SW PT
- STROKE / GERI CONS
- STROKE NURSE
- GP
- support workers
- ? Neurorehab manager / Stroke case manager
- IHF Stroke Support coordinators
- Community Rehabilitation Assistants
- StrokeLINK or similar service
- key workers

Stakeholders | 10.06.2022

Other

Politicians

MAINSTREAM MEDIA

National Advocacy Service for People with Disabilities

Sage

NDA

3rd sector

ihf

abii

headway

aphasia ireland

Croí

Alone

? return to work services

? social welfare etc. ombudsman

inclusion Ireland

Easy to Read Ireland

Crown

Quest BI

ETB

age action

PPI

PWA

caregivers

aphasia advocate networks - e.g. via UCC

IHF groups

Dissemination formats | 10.06.2022

content	academic papers	accessible PPT	animation	policy brief	Presentation
• SLT survey<br>• OT / PSY survey	X				X
• SEA CoP formation, survey	X		X		X
• PPI, Co-design methods<br>"Outside the box"	X				X
• Qual interviews	?				X
• Overall recommendations	X	X	X	X	X

Today

1. Re-visit your Aphasia SPC reflections.

2. What should be prioritised / where to start?

3. Stakeholders & Dissemination

4. **Close out sessions.**

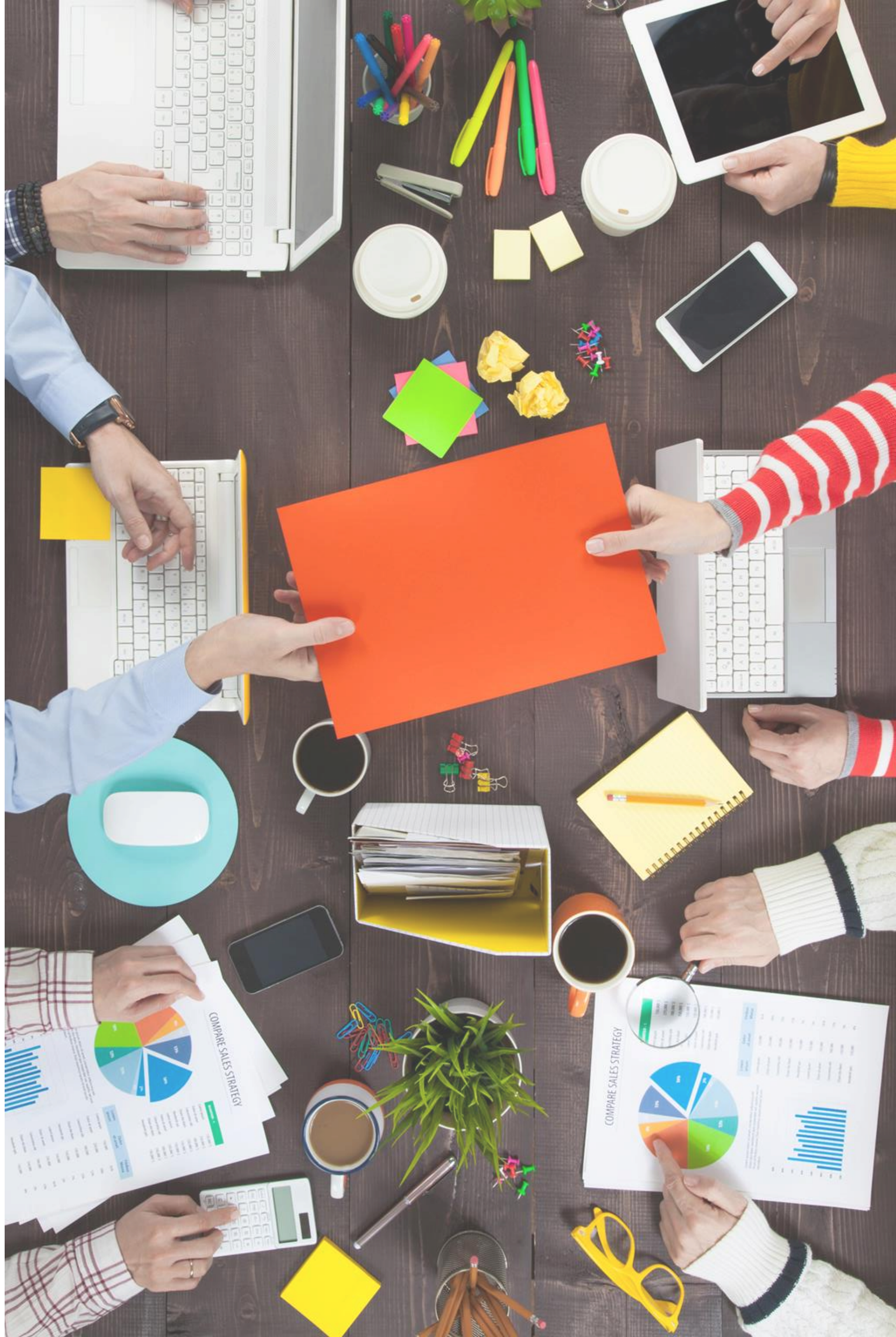

THANK YOU!

Please get in touch if you have any questions:

Molly.manning@ul.ie

0877740987

https://miro.com/app/board/uXjVO3TVTfk=/?share_link_id=798688146033
