# Supplementary material for: Advancing aphasia psychological care in Ireland: A participatory study with people with aphasia and clinicians
Source: PLoS One. 2026 Apr 20;21(4):e0331427. doi: 10.1371/journal.pone.0331427 (PMC13095103; doi:10.1371/journal.pone.0331427)
Supplement: Attachment — Submitted filename: Response to Reviewers.docx [file pone.0331427.s004.docx]

**RESPONSE TO REVIEWERS**

|  | General points in email |  |
| --- | --- | --- |
| 1 | Please ensure that your manuscript meets PLOS ONE's style requirements, including those for file naming. The PLOS ONE style templates can be found at  <https://journals.plos.org/plosone/s/file?id=wjVg/PLOSOne_formatting_sample_main_body.pdf> and  <https://journals.plos.org/plosone/s/file?id=ba62/PLOSOne_formatting_sample_title_authors_affiliations.pdf> | Thank you. We have reviewed the PLOS ONE style requirements and ensured that our manuscript and all associated files comply with these guidelines. |
| 2 | Thank you for stating the following financial disclosure: This work was supported by Seed Funding from the Faculty of Education and Health Sciences, University of Limerick, awarded to Dr Molly Manning in 2021.  Please state what role the funders took in the study. If the funders had no role, please state: "The funders had no role in study design, data collection and analysis, decision to publish, or preparation of the manuscript." If this statement is not correct you must amend it as needed. Please include this amended Role of Funder statement in your cover letter; we will change the online submission form on your behalf. | Thank you – this is now stated in the cover letter above. |
| 3 | We noted in your submission details that a portion of your manuscript may have been presented or published elsewhere. Please clarify whether this publication was peer-reviewed and formally published. If this work was previously peer-reviewed and published, in the cover letter please provide the reason that this work does not constitute dual publication and should be included in the current manuscript. | This work was not previously formally published. |
| 4 | We note that you have indicated that there are restrictions to data sharing for this study. For studies involving human research participant data or other sensitive data, we encourage authors to share de-identified or anonymized data. However, when data cannot be publicly shared for ethical reasons, we allow authors to make their data sets available upon request. For information on unacceptable data access restrictions, please see <http://journals.plos.org/plosone/s/data-availability#loc-unacceptable-data-access-restrictions>.  Before we proceed with your manuscript, please address the following prompts:  a) If there are ethical or legal restrictions on sharing a de-identified data set, please explain them in detail (e.g., data contain potentially identifying or sensitive patient information, data are owned by a third-party organization, etc.) and who has imposed them (e.g., a Research Ethics Committee or Institutional Review Board, etc.). Please also provide contact information for a data access committee, ethics committee, or other institutional body to which data requests may be sent.  b) If there are no restrictions, please upload the minimal anonymized data set necessary to replicate your study findings to a stable, public repository and provide us with the relevant URLs, DOIs, or accession numbers. Please see <http://www.bmj.com/content/340/bmj.c181.long> for guidelines on how to de-identify and prepare clinical data for publication. For a list of recommended repositories, please see <https://journals.plos.org/plosone/s/recommended-repositories>. You also have the option of uploading the data as Supporting Information files, but we would recommend depositing data directly to a data repository if possible.  Please update your Data Availability statement in the submission form accordingly. | A de-identified version of the final clinician slide deck, incorporating all contributions and the results of iterative thematic analysis, is now provided in Supplementary File S2. Contributions from the PPI meeting were not subjected to formal thematic analysis; beyond grouping and synthesising inputs using PLA techniques, they served to inform study progress at a meta-level.  The Data Availability statement has been updated accordingly. |

|  | **Additional Editor Comments** | **Changes made** |
| --- | --- | --- |
| 1 | 1. Significance of the Research This manuscript addresses a critical and understudied gap in clinical practice: the absence of coordinated, context-specific psychological care for individuals with aphasia within the Irish healthcare system. The well-documented association between aphasia and adverse psychosocial outcomes, including depression, anxiety, and social isolation, underscores the urgency of this issue. The study's commitment to stakeholder co-design—centering the perspectives of people with aphasia (PWA) and interdisciplinary clinicians—aligns directly with PLOS ONE's mission to publish applied health research with tangible real-world impact. | Thank you for recognising the significance of this research and its potential impact. We greatly appreciate your thoughtful feedback and support. |
| 2 | The relevance of this work is further enhanced by its grounding in the distinctive Irish healthcare context, with explicit reference to pertinent national policies such as the National Stroke Strategy 2022–27 and the Sláintecare reform program. By systematically linking local service deficiencies (e.g., constrained access to psychological services, inconsistent mood screening protocols) to broader implementation science challenges, the study occupies a unique niche in the extant literature, which frequently focuses on larger healthcare systems while neglecting smaller, contextually specific environments like Ireland. | Thank you. |
| 3 | Notwithstanding these strengths, **the manuscript would benefit from a more explicit articulation of the transferability of its findings beyond the local context**. For instance, the identified principles of a "unified vision" and "flexible referral pathways" may yield valuable lessons for other jurisdictions grappling with comparable resource limitations or decentralized service delivery models. Elaborating on this dimension would significantly bolster the manuscript's appeal to PLOS ONE's international readership and reinforce its broader scholarly significance. | The following paragraph has been added to the Conclusion section:  “*While this study focused on Ireland, many of the structural barriers identified are shared across healthcare systems that experience regional fragmentation, limited psychological resources, or gaps in community integration. The principles developed through this participatory process, such as the importance of a shared vision, stepped care design, flexible referral routes, and integrated informal support, are likely to be applicable in other settings. In particular, jurisdictions with underdeveloped psychological support for people with aphasia or low interdisciplinary coordination may find value in the stakeholder-led methods and the emerging APC framework. The participatory implementation approach used here may also be adapted to other conditions or contexts, supporting locally relevant intervention planning without assuming direct replication.*”  A new sentence has also been added to the conclusion section of Abstract: *“The participatory implementation approach may be adapted to other conditions or contexts to support locally relevant intervention planning.*” |
| 4 | The manuscript's principal innovative contribution lies in its methodological approach: the deliberate integration of Participatory Learning and Action (PLA) tools with Normalization Process Theory (NPT) to structure stakeholder engagement. While both PLA and NPT have been employed independently within aphasia research, their synergistic combination here is both deliberate and purposeful. PLA facilitates inclusive knowledge co-creation with PWA, a population often facing significant barriers to research participation, while NPT supplies a robust theoretical framework to translate these insights into implementable strategies. This dual methodology effectively addresses a recognized limitation in prior participatory studies, which often lack explicit connections to implementation science theory. | Thank you. |
| 5 | The proposition of a "level 0" within the stepped care model—formally acknowledging the informal, non-clinical support systems upon which PWA frequently rely (e.g., peer networks, self-management strategies)—constitutes another substantive contribution. Although the Personal Recovery paradigm has previously informed aphasia care, the manuscript's focus on operationalizing "level 0" as an integral component of stepped care, rather than a peripheral consideration, represents a novel advancement. This approach validates the agency of PWA and recognizes that recovery processes often originate outside formal clinical settings—a conceptual shift with potential to reconfigure stepped care design for neurogenic disorders beyond aphasia. | Thank you. |
| 6 | **To fully realize this innovative potential, the manuscript should elaborate on the functional interplay between "level 0" and established care levels.** Specifically, it should address operational considerations such as: What mechanisms would trigger referrals from level 0 to level 1? How would engagement or outcomes at level 0 be assessed in clinical practice? Providing concrete examples or stakeholder-derived operational guidelines would substantially enhance the credibility of "level 0" as an actionable clinical concept. | The following content has been added to the Discussion section:  “*The inclusion of a conceptual Level 0 reflects stakeholder recognition that recovery often begins before and outside formal service engagement. Examples include peer support, informal befriending, social prescribing, and community activities that promote psychological wellbeing. These activities may be accessed directly, without referral, and may offer early benefits such as reduced isolation or increased confidence. While Level 0 does not involve clinical intervention, it may serve as a platform for identifying individuals who would benefit from more structured support.*    *Engagement and outcomes at Level 0 could be monitored through attendance tracking, simple mood or wellbeing self-ratings, informal goal setting, or feedback from peers or family members. These low-burden and community-engaged approaches reflect the ethos of Level 0 and tend to be viewed by stakeholders as acceptable, valid and feasible in community-based and / or community-led practice (NEW REFALSO ADDED – Chutiyami et al 2025). Specific methods may include: (i) attendance records; (ii) pictorial mood scales or visual analogue tools co-developed with people with aphasia; (iii) Goal Attainment Scaling adapted for supported conversation; (iv) structured or informal feedback from peers, carers, or group leaders; and (v) brief reflective conversations around personally meaningful goals.*” |
| 7 | 2. Research Methods The methodological framework is generally robust and adheres to PLOS ONE's standards for qualitative inquiry, evidenced by compliance with the Standards for Reporting Qualitative Research (SRQR) and clear documentation of ethical approval (University of Limerick, 2021_12_19). The decision to conduct separate workshops for clinicians and PWA, followed by structured cross-group knowledge integration, is particularly commendable. This approach enables candid discourse within each stakeholder group while ensuring the systematic incorporation of diverse perspectives—a crucial safeguard against tokenistic patient and public involvement. | Thank you. |
| 8 | However, three significant limitations require remediation to satisfy PLOS ONE's rigor requirements:  **Sample Size and Diversity** The participant cohort (6 clinicians, 3 PWA), while permitting in-depth participatory dialogue, exhibits limited diversity across critical dimensions. The clinician sample overrepresents senior, specialized roles (e.g., Senior Speech and Language Therapists, Clinical Neuropsychologists) while underrepresenting frontline primary care providers (e.g., General Practitioners, community nurses) who frequently function as gatekeepers to aphasia psychological care. Regarding PWA participants, all were working-aged individuals with long-term aphasia (10–15 years post-stroke), thereby excluding those with acute presentation or non-stroke etiologies (e.g., traumatic brain injury)—populations with distinct psychological needs. The authors should explicitly acknowledge these limitations and, where feasible, supplement the discussion with insights from relevant literature concerning underrepresented groups to better contextualize their findings. | Thank you. The methodological limitations section has been updated to reflect these important insights.  “There are several important methodological limitations, and the findings should be appraised accordingly. This study provides some initial ideas generated through participatory research discussions with a small number of aphasia clinicians and PPI contributors. It is likely that these are not comprehensively representative of all services, settings, and experiences. The clinician group predominantly represents senior, specialised roles, whilst underrepresenting frontline primary care providers such as General Practitioners and community nurses. The aphasia group comprised only working-aged individuals living with aphasia for 10 years and longer, thereby excluding those with acute presentations and non-stroke aetiologies of aphasia, also populations with distinct psychological needs. This is particularly salient given that the agreed scope and focus of coordinated APC in this study ideally encompasses non-stroke aetiologies as well. It is important that the insights generated are appraised accordingly and that future research draws on sustained engagement with a wider range of interest holders, including families of people with aphasia, policy makers, and a wider range of key gatekeepers to mental health support in Ireland. Future research should also explore opportunities for combined meetings with the clinician co-researchers, aphasia PPI contributors and other interest holders.” |
| 9 | **Transparency in Data Analysis** Although the manuscript describes the employment of PLA techniques (e.g., sticky note brainstorming, card-sorting) and NPT operationalization, it lacks sufficient detail regarding systematic analytical procedures. Critical methodological information is absent, including: The specific coding methodology applied to qualitative data (e.g., inductive versus deductive approaches); Measures implemented to ensure coding reliability (e.g., independent coding by multiple researchers, consensus-building procedures); The precise application of NPT constructs to interpret emergent themes. The inclusion of a supplementary table or detailed paragraph elucidating the analytical workflow would satisfy PLOS ONE's requirement for reproducible qualitative methodologies. | Thank you for this excellent observation. The following content has been added to the Methods section, which we hope strengthens the clarity and rigour of the methodological reporting.  “Although NPT constructs guided the discussion prompts, the knowledge generated through PLA techniques across clinician meetings was subjected to an iterative thematic analysis process (58). The first author led this analysis, with continuous scrutiny and collaborative input from co-authors during meetings and through co-creation of the final report. Following each clinician meeting, all contributions and virtual ‘stickies’ were systematically reviewed to identify similarities, differences, and emerging insights. These informed the development of preliminary themes and descriptors that captured patterns of meaning relevant to the research questions. A summary of these evolving themes, supported by all contributions, was prepared and presented at the start of each subsequent meeting, functioning as a de facto data analysis clinic. This approach enabled member checking and strengthened trustworthiness (59). The process was highly iterative, with themes refined through ongoing discussion across meetings and during report preparation. To enhance reflexivity, the first author maintained detailed notes throughout, documenting emerging interpretations, positionality, and potential biases (60). A de-identified version of the final clinician slide deck—incorporating all contributions and the results of this iterative thematic analysis—is provided in Supplementary File S2. Contributions from the PPI meeting were not formally analysed thematically; instead, they were grouped and synthesised using PLA techniques to inform study progress at a meta-level.” |
| 10 | Data Availability The current data availability statement ("No - some restrictions will apply") fails to comply with PLOS ONE's policy mandate that authors "make all data underlying the findings described fully available, without restriction" (except where legally or ethically justified). The authors must provide explicit clarification regarding: The specific categories of data subject to restrictions (e.g., raw verbatim comments to protect participant anonymity); Accessibility procedures for non-restricted data (e.g., de-identified thematic summaries, workshop protocols); The ethical or legal rationale underpinning any restrictions (e.g., specific informed consent agreements with PWA). A comprehensive and policy-compliant data availability statement is mandatory for resubmission. | As above, a full summary of clinician contributions and iterative analysis is supplemented. Data availability statement has been updated. |
| 11 | 3. Writing and Presentation The manuscript demonstrates sound organizational structure and accessibility, with a coherent progression from background through methods, results, and discussion. Notable strengths include: clear explication of technical terminology (e.g., "stepped care," "PLA") appropriate for interdisciplinary readership; strategic incorporation of direct quotations from PWA, anchoring findings in lived experience; consistent alignment between stated research objectives and presented results. | Thank you |
| 12 | Nevertheless, several revisions would enhance clarity and professional presentation:  Redundancy Several key points (e.g., the necessity for enhanced clinician training, barriers to interdisciplinary collaboration) are repetitively addressed across the introduction, results, and discussion sections. Maybe the authors could further streamline the similar expressions to improve readability. | Thank you for this suggestion. We have edited the manuscript to reduce redundancy regarding clinician training and collaboration points that were previously repeated throughout. All changes are indicated using tracked edits. |
| 13 | **Table Design** **Tables 1 (Operationalisation of NPT Constructs) and 2 (Sequence and Focus of Meetings) require enhanced detail to ensure methodological reproducibility:** **Table 1** should incorporate concrete illustrations of how NPT constructs guided discussions (e.g., "Context: Prompted clinicians to examine how HSE funding constraints impact APC delivery"); | Table 1 has been revised to provide additional detail on how NPT sub-constructs informed discussions. However, the discussions were intentionally broad and exploratory, spanning both aphasia and clinician groups, rather than being tied to specific prompts. As such, adding more granular examples would risk misrepresenting the open and integrative nature of these conversations. |
| 14 | **Table 2** should rectify formatting inconsistencies (e.g., truncated prompts) and incorporate temporal markers (e.g., "April 2022: Clinicians 1 workshop") to clarify the research timeline. | Thank you for your helpful comment.  We have revised Table 2 to address the formatting inconsistencies and ensure clarity.  The updated table now provides a clearer and more coherent representation of the research timeline and content discussed at each stage. |
| 15 | Reference Consistency The reference list contains several technical inaccuracies: Baker (20) (cited in Section 1.46) is omitted from the references; Citations for Leamy et al. (2011) and Manning et al. (2019) appear in incorrect sequence. Rectifying these errors is necessary to ensure compliance with PLOS ONE's bibliographic standards. | Thank you. We have thoroughly reviewed the references and made the necessary revisions, and we hope that all concerns raised by the reviewers have now been fully addressed. |
| 16 | 4. Conclusion This manuscript constitutes a valuable contribution to aphasia care research through its principled centering of stakeholder perspectives and its systematic linkage of participatory insights with actionable implementation strategies. Its focused examination of the Irish healthcare context addresses a significant literature gap, while its methodological integration of PLA and NPT offers a replicable framework for future co-design studies**. Provided the authors address the identified concerns regarding sample diversity, analytical transparency, and presentational clarity**, the revised manuscript will fully satisfy PLOS ONE's academic standards and serve as an important resource for clinicians, policymakers, and researchers dedicated to enhancing psychological support for people with aphasia.  To facilitate efficient re-evaluation, the authors should prioritize the following revisions: | Thank you. We hope that our revisions have satisfactorily addressed all concerns raised by the reviewers. |
| 17 | 1) Elaborate the discussion regarding the adaptability of findings to other healthcare contexts to enhance international relevance. | As noted above, we have added a final sentence to the conclusion of the abstract and included a new paragraph in the main Conclusion section. |
| 18 | 2) Provide concrete operational specifications for the "level 0" stepped care model, including potential referral triggers and assessment approaches. | As noted above, we have added new content to the Discussion section. |
| 19 | 3) Incorporate a comprehensive description of the data analysis workflow, detailing coding methodologies and reliability measures. | As above. |
| 20 | 4) Revise the data availability statement to achieve full compliance with PLOS ONE policies, explicitly clarifying restrictions and access procedures. | This has been updated. |
