# Supplementary material for: Advancing aphasia psychological care in Ireland: A participatory study with people with aphasia and clinicians
Source: PLoS One. 2026 Apr 20;21(4):e0331427. doi: 10.1371/journal.pone.0331427 (PMC13095103; doi:10.1371/journal.pone.0331427)
Supplement: Attachment — Submitted filename: Response_to_Reviewers_auresp_2.docx [file pone.0331427.s005.docx]

**RESPONSE TO REVIEWERS**

|  | **Reviewer 2 Comments** | **Changes made** |
| --- | --- | --- |
| 1 | In the “Knowledge generation and synthesis” subsection, it might be helpful to add a brief sentence explaining how the iterative thematic analysis complemented the NPT-guided discussion prompts. This would further clarify your analytical strategy for readers. | Thank you for this suggestion.  We have overhauled the relevant content in the Knowledge Generation and Synthesis section as follows:  “Initially, NPT informed the development of questions related to clinicians’ sense‑making, their perceptions and experiences on current care (Differentiation, Individual Specification). After each clinician meeting, all contributions and virtual ‘stickies’ were systematically reviewed to identify similarities, differences, and emerging insights. These informed the development of preliminary themes and descriptors capturing patterns of meaning relevant to the research questions.  A summary of these evolving themes, supported by all contributions, was presented at the start of each subsequent meeting, functioning as a de facto data analysis clinic This approach enabled member checking and strengthened trustworthiness (59). The emerging themes guided further discussion about principles of good care should entail, and the perceived value and differences compared with current practice (Communal Specification and Appraisal).  The knowledge generated through these discussions was again synthesised and thematically organised. Refined themes were subsequently presented to guide dialogue on stepped‑care design and priorities for implementation (Cognitive Participation and Collective Action constructs). Following the second clinician meeting, these evolving themes were also presented to PPI contributors, whose input was incorporated alongside clinician comments to validate findings and inform subsequent meetings.  ” |
| 2 | Regarding the discussion on creative therapies, the evidence is currently described as “equivocal.” To maintain a strong yet perfectly balanced recommendation, you might consider phrasing that acknowledges the developing evidence base while underscoring the stakeholder-endorsed rationale for its inclusion. | Thank you for this helpful suggestion – we have simplified the wording as follows:  “Creative and mind-body interventions were of interest across both research groups. This is in keeping with a growing body of research on alternative and creative modality psychological interventions (71), which may be advantageous in the context of aphasia, given their minimal language load  (72).” |
|  | **Reviewer 3 Comments** | **Changes made** |
| 1 | In the Methods section, the authors have expanded the description of the analytic process. This revision is helpful. However, it is still not fully clear whether the thematic analysis was primarily inductive, deductive (guided by NPT), or hybrid. Please specify this explicitly. | Thank you for these helpful comments.  The Knowledge Generation and Synthesis section has been revised for clarity relating to (1) inductive analysis process; (2) independent coding by first author; (3) analysis of PPI contributions. A new sentence has also been added to the Methodological Critique relating to the imbalance in analytical weight across PPI and clinician contributions.  REVISED TEXT IN KNOWLEDGE GENERATION & SYNTHESIS:  “Although NPT constructs guided the discussion prompts, the knowledge generated through PLA techniques was analysed using an iterative thematic analysis process (58). The first author led this process, coding all material, with continuous scrutiny and collaborative input from co-authors during meetings and through co-creation of the final report. The analysis was inductive and highly iterative, with themes refined through ongoing discussion.  Initially, NPT informed the development of questions related to clinicians’ sense‑making, their perceptions and experiences on current care (Differentiation, Individual Specification). After each clinician meeting, all contributions and virtual ‘stickies’ were systematically reviewed to identify similarities, differences, and emerging insights. These informed the development of preliminary themes and descriptors capturing patterns of meaning relevant to the research questions.  A summary of these evolving themes, supported by all contributions, was presented at the start of each subsequent meeting, functioning as a de facto data analysis clinic This approach enabled member checking and strengthened trustworthiness (59). The emerging themes guided further discussion about principles of good care should entail, and the perceived value and differences compared with current practice (Communal Specification and Appraisal).  The knowledge generated through these discussions was again synthesised and thematically organised. Refined themes were subsequently presented to guide dialogue on stepped‑care design and priorities for implementation (Cognitive Participation and Collective Action constructs). Following the second clinician meeting, these evolving themes were also presented to PPI contributors, whose input was incorporated alongside clinician comments to validate findings and inform subsequent meetings.  To enhance reflexivity, the first author maintained detailed notes, documenting emerging interpretations, positionality, and potential biases (60). A de-identified version of the final clinician slide deck—incorporating all contributions and the outputs of the iterative thematic analysis—is provided in Supplementary File S2. Feedback from this final stage was thematically summarised, forming the basis of the final reported themes.”  REVISED TEXT IN METHODOLOGICAL CRITIQUE:  “Furthermore, future participatory research in this area should give greater analytic weighting to knowledge generated with people with lived experience, in order to address the imbalance in the present study, which drew more heavily on clinician contributions.” |
| 2 | In the same section, the manuscript states that the first author led the coding process with ongoing scrutiny from co-authors. It would strengthen methodological transparency to clarify whether any independent coding of raw data was conducted by more than one researcher before consensus discussions took place. |  |
| 3 | Also in the Methods section, the manuscript notes that PPI meeting data were not formally analysed thematically but were grouped at a meta-level. Please provide a clearer methodological justification for this difference in analytic treatment and explain how this approach ensured that PPI contributions were given equal analytic weight. |  |
